# Smooth dynamic T_2_^*^ mapping in fMRI based on a novel, total variation-minimizing algorithm for efficient multi-echo BOLD time series denoising with high signal-to-noise and contrast-to-noise ratios

**DOI:** 10.3389/fnins.2025.1544748

**Published:** 2025-10-01

**Authors:** Jan Michálek, Michal Mikl

**Affiliations:** ^1^Faculty of Informatics, Centre for Biomedical Image Analysis, Masaryk University, Brno, Czechia; ^2^Multimodal and Functional Imaging Laboratory (MAFIL), Central European Institute of Technology—CEITEC, Masaryk University, Brno, Czechia

**Keywords:** BOLD signal restoration, denoising, multi-echo fMRI, inexact ADMM, quantitative fMRI, T_2_^*^ mapping, total variation minimization

## Abstract

**Introduction:**

This report deals with advanced processing of blood oxygenation-dependent (BOLD) functional magnetic resonance imaging (fMRI) signals. It does not address functional characteristics of the human cortex, such as functional connectivity. fMRI is based on measurement of BOLD variations of transverse relaxation time T_2_^*^ or *T_2_*. T_2_^*^ or *T_2_* can be calculated when multiple echoes of the MRI signal are recorded and may be more resistant to artifacts or better characterize tissue properties than the echoes themselves.

**Objectives:**

To develop a robust-to-noise algorithm for dynamic T_2_^*^ mapping from a three gradient-echo (GRE) signal, allowing exploration of the potential of quantitative T_2_^*^ mapping.

**Methods:**

fMRI resting-state and block-design visual task three-echo data were acquired from nine healthy volunteers. A significant problem in multi-echo T_2_^*^ fitting is the noise in the echoes. The majority of BOLD-denoising methods first pinpoint some source of noise and subsequently remove the respective noise time series. We instead first postulated that the blood oxygenation changes smoothly and consequently developed a state-of-the-art denoising algorithm that minimizes total variation (TV), enforcing smoothness in the processed BOLD echoes while preserving local temporal signal means. To ensure that calculated T_2_^*^ time courses are also smooth, they were estimated from TV-denoised echoes. We used a denoising approach initially proposed by Professor Stanley Osher for two-dimensional (2D) images that has been very successful, most prominently in space research, where it enabled the reconstruction of the first-ever image of a black hole. To our knowledge, Osher’s approach has so far not been used elsewhere for the denoising of one-dimensional fMRI time series.

**Results:**

Signal-to-noise and contrast-to-noise distributions of the denoised echoes, as well as of the T_2_^*^ time series, were superior to those obtained by the current fMRI denoising methods (3dDespike, *tedana*, NORDIC). The denoised echoes and the T_2_^*^ time courses match the shape of the theoretical hemodynamic function much better than previous results.

**Conclusion:**

The TV-minimizing fMRI time series denoising algorithm yields denoised echoes of unprecedented quality, enabling estimation of smooth, dynamic T_2_^*^ maps, i.e., a transition from qualitative-only fMRI echoes to fMRI signals endowed with time units.

## 1 Introduction

Functional magnetic resonance imaging (fMRI) is broadly used in neuroscience research and, to some extent, in clinical applications. The classical fMRI approach is based on the measurement of blood oxygenation level-dependent (BOLD) signal changes and analysis of temporal signal fluctuations. Paramagnetic deoxyhemoglobin in venous blood is a naturally occurring contrast agent for magnetic resonance imaging (MRI). Ogawa et al. (1990) demonstrated *in vivo* images of brain microvasculature with image contrast reflecting the blood oxygen level by accentuating this agent’s effects through gradient-echo techniques in high magnetic fields.

Because the BOLD signal units are arbitrary, the interpretation of single-echo data is limited to relative changes of the BOLD signal utilizing some statistic (typically t-values). Therefore, the results are affected by the signal-to-noise ratio, i.e., also by the hardware configuration used and by the acquisition protocol.

**Figure 1:**
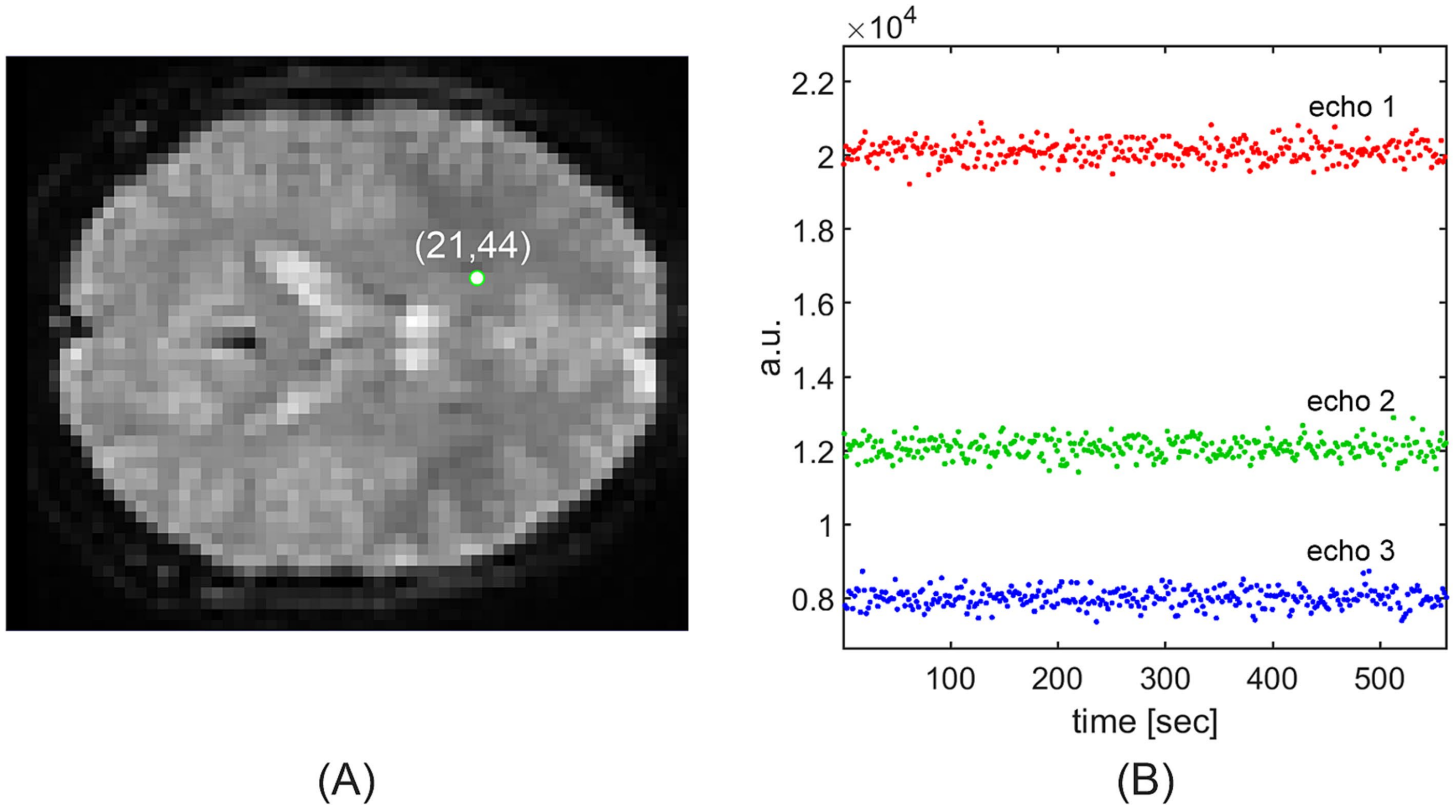
Time courses of three BOLD signal echoes **(B)** at the green-marked voxel **(A)** of a resting-state multi-echo fMRI series comprising 312 frames (data from sub-01). The BOLD signal was sampled with the repetition time of TR = 1,800 ms. As invasive measurements in animals show that the true BOLD signal is smooth, the sampled fMRI signal should also be smooth; however, it fluctuates strongly and needs to be denoised. Without denoising, an estimate of the exponential time constant from the three noisy echoes would result in a strongly oscillating, rather than smooth, T_2_^*^ time course.

Within a few years after the discovery of the BOLD effect, it was recognized in gradient-echo and spin-echo experiments that the BOLD contrast is echo-time dependent (Bandettini et al., 1994). In multi-echo fMRI, several echoes are acquired after a single excitation during one sampling interval, which enables combining data from particular echoes to enhance BOLD contrast sensitivity in each voxel. Moreover, there is a possibility to calculate T_2_^*^ from the echoes and subsequently analyze data quantitatively. Such an approach might increase the robustness and the reproducibility of the fMRI analysis. Unfortunately, the acquisition protocol used for multi-echo fMRI is not identical to the standard relaxometric protocol. The BOLD signal measurements are noisy, Figure 1, and simple exponential fitting amplifies measurement noise [Michálek et al., 2019, Equations (12–14)]. Therefore, more robust algorithms are needed to fully exploit the potential of quantitative multi-echo data-based T_2_^*^ analysis.

One of the earliest studies (Gati et al., 1997) on multi-echo fMRI experiments estimated the relaxation rate R_2_^*^ (the reciprocal of T_2_^*^) using eight multi-shot gradient echoes within a single slice to fit. A series of 40 consecutive images was acquired while the task stimulus toggled between periods of dark (baseline) and flashing (activation) states. Five images were collected during each period. The R_2_^*^ values were not estimated voxelwise but within regions of activated visible veins (referred to as “vessels”) and cortical gray matter (“tissue”) over which the signal values were averaged.

Posse et al. (n.d.) argued that BOLD contrast fMRI suffers from several limitations, among others lack of quantitation. To address this issue, they quantitatively measured T_2_^*^ signal relaxation during visual and olfactory stimulation using a pulse sequence named Turbo-PEPSI that acquired 12 echo planar images with echo times (TE) ranging from 12 to 228 ms. Reconstructed magnitude images were fitted with single exponential lineshapes of the form: $S=S_{0}\exp(-\mathrm{TE}/{T_{2}}^{*})$ . When fitting the relaxation time course data, it became apparent that activation maps based on T_2_^*^ displayed higher sensitivity than those obtained with conventional EPI (Posse, 2012).

Transverse relaxation time (T_2_^*^) and initial signal intensity (*S_0_*) mapping using a single-shot EPI sequence were presented in the study by Speck and Hennig (1998). *S_0_* changes are a measure of the inflow sensitivity to stimulation, while T_2_^*^ is a direct measure of the BOLD-related signal change. Mappings were performed in three horizontal slices. Overall, 120 sets of 24 or 18 images (three slices, eight or six echo images) were acquired with a repetition time (TR) of 3 s. The stimulus was turned off and on for 30 s, respectively. Two data versions (eight echo images with a matrix size of 64 × 32 or six echoes with a 64 × 64 matrix) were acquired. For every set of eight (or six, respectively) echo images, the parameter maps were calculated from a pixel-by-pixel least squares fit of a monoexponential to the data points. The authors only show the time courses of average *S_0_* and T_2_^*^ values within areas where a correlation coefficient with a boxcar function corresponding to visual stimulation was greater than 0.3. The time courses in individual voxels were not provided.

Posse et al. (1999) published a comprehensive analytical treatment of fMRI contrast enhancement in which knowledge of T_2_^*^ plays an important role. First, they came to a fundamental result: the contrast for a single-echo BOLD signal has the maximum value when the echo time equals T_2_^*^. This cannot be generally satisfied for all voxels since only a single time point close to the expected BOLD optimum is measured. Additional functional information can be obtained by sampling multiple echo times in a single shot and combining the datasets thus obtained. Posse et al. (1999) analyzed different types of preprocessing of the multi-echo datasets:

- Summation of datasets acquired at different echo times.
- A weighted summation of the datasets acquired at different echo times. The weights depend on expected T_2_^*^ values of individual voxels as well as on the echo times.
- Curve fitting to quantitate changes in T_2_^*^ and *S_0_*.

Posse et al. (1999) derived formulas proving that the initially increasing contrast of simple summation decreases again after a T_2_^*^-dependent peak is passed. Weighted summation of the datasets should yield the highest contrast improvement, closely followed by T_2_^*^ fitting, both of which saturate with an increasing number of measured echoes.

Hagberg et al. (2002) derived a numerical method referred to as NumART2^*^ that allowed for rapid whole-brain mapping of T_2_^*^. The method numerically approximates the area under the exponential decay curve by replacing the exponential with straight lines with their endpoints at the echo sample points. Based on this approximation, T_2_^*^ is directly calculated as a linear combination of images obtained at three or more different echo times. The T_2_^*^ estimation from an fMRI task multi-echo signal is very fast, but the time courses shown in activated voxels are extremely noisy (signal jumps ~ 50%) despite T_2_^*^ being estimated from a large number (eight) of echoes.

For resting-state fMRI data, Kundu et al. (2012) published a different multi-echo approach in which the blood oxygenation-induced temporal changes in the echoes are separated from the inflow-dependent ones based on the observation that BOLD signal changes exhibit linear dependence on the echo time while the proton-density (inflow)-dependent ones do not. Based on two statistics named rho and kappa, it can be distinguished for each brain voxel whether the TE-dependence or the inflow dependence prevails. Ensuing independent component analysis (ICA) labels components deemed TE-independent that are regressed out of the voxel time series as noise, thus increasing the signal-to-noise ratio. The approach was extended to task-based fMRI analyses in Kundu et al. (2017).

Weighted summation of the multi-echo datasets discussed in Posse et al. (1999), later referred to as “optimal combination,” was implemented by the authors of DuPre et al. (2021) as a BOLD signal preprocessing option and is freely available as part of their Python library named *tedana* (script *t2smap.py*), which is intended for denoising of multi-echo fMRI data.

Heunis et al. (2021) compared six different strategies used to combine multi-echo fMRI data: a single-echo time series (based on echo 2), the real-time T_2_^*^-mapped time series (T2^*^FIT), and four combined time series (T_2_^*^-weighted, tSNR-weighted, TE-weighted, and a new combination scheme termed T2^*^FIT-weighted). They evaluated the performance in terms of several metrics such as temporal signal-to-noise ratio (tSNR), task activity effect size, region of interest (ROI)-based temporal percentage signal change (tPSC), functional contrast, and temporal contrast-to-noise ratio (tCNR). They recommended the use and continued exploration of their T2^*^FIT method for offline task-based and real-time region-based fMRI analysis because the T2^*^FIT time series consistently yielded the largest offline effect size measures, and real-time ROI-based functional contrasts and temporal contrast-to-noise ratios. The only drawback of the T2^*^FIT time series they observed was the decrease in tSNR. For this reason, they advised further research to mitigate the decreased tSNR of the T2^*^FIT time series. Their recommendation is particularly important regarding the total variation (TV)-based T_2_^*^ mapping algorithm described in our study, which—as will be shown in Chapter 3—does increase, not decrease, tSNR.

### 1.1 Noise and denoising in fMRI measurements

The survey above shows that noise in multi-echo fMRI measurements distorts input data to the T_2_^*^ fitting equations and is detrimental to T_2_^*^ mapping. Multiple sources contribute to fMRI noise (Liu, 2017): background noise, noise in the magnetization term, and noise in the relaxation term. Some noise components in the magnetization term and in the relaxation term are caused by physiological processes such as subject motion, cardiac pulsations, and respiratory activity. Noise related to periodic processes (cardiac or respiratory) can be effectively removed by spectral analysis of the fMRI signal and matched band-pass filtering. Other advanced algorithms for physiological noise separation, like that of Kundu et al. (2012, 2017), first perform ICA. Then, the echo-time dependence of independent fMRI components is exploited to distinguish between BOLD-like components (i.e., those components with voxel amplitudes that show a linear dependence on echo time) and non-BOLD-like noise components that do not exhibit a strong linear dependence. As a final step, the non-BOLD components are regressed out.

The fMRI background noise reflects the contributions of sources that are independent of the signal of interest, e.g., thermal noise arising from the thermal agitation of charge carriers in both the subject and the MRI system electronics, radio frequency (RF) spikes due to intermittent mechanical contacts between metal components, and spurious RF noise from the environment. The background noise term is present even if there is no activity-related signal of interest and can be measured simply by acquiring the data without exciting any magnetization.

Random uncorrelated background noise appears in the measured fMRI signal as spikes of random magnitude at random time instants. Isolated spikes cannot be fully removed by classical denoising methods like frequency filtering, ICA, or principal component analysis (PCA). The Fourier spectrum of a single spike contains all frequencies with the same magnitude and, therefore, cannot be separated from the BOLD signal of interest by any type of frequency filtering. Frequency filters remove only some of the frequencies (low-pass, high-pass, band-pass, etc.); hence, some frequency components of the spike will be retained. A similar argument holds for component-based signal-noise separation, such as PCA or ICA. In PCA, the principal components are eigenvectors of the signal autocovariance matrix, i.e., they are a time series. PCA filtering removes principal components (eigenvectors), whose associated eigenvalues are below a certain threshold, from the time series expansion, whereas eigenvectors related to large eigenvalues are retained. Again, a single spike is expanded as a linear combination of all the eigenvectors; therefore, deleting only some of the eigenvectors from the expansion cannot completely remove the spike. Similarly, independent components are also whole time series, not isolated spikes.

Contrary to that, TV-based denoising removes isolated signal peaks *by design*. Total variation of a time series is simply the sum, over the whole series, of all *absolute* signal *changes* from one time instant to the next one. If the start and the end of a time series are connected by a monotonically increasing or decreasing signal, the TV is the absolute value of the difference between the start and the end. When the fMRI signal is contaminated by random spikes, the true monotonic signal is disguised in a sawtooth-like measured time series with the saw teeth at the spike locations. The sum of absolute differences (TV) of the sawtooth wave is, of course, higher than that of the monotonic true signal, so minimization of the signal TV while keeping it close to the measured signal restores the smooth original, increasing or decreasing, signal. Thus, minimization of TV—contrary to classical denoising approaches—effectively removes spurious noise (spikes). Our aim was to develop an algorithm that would yield T_2_^*^ time courses more faithful to the canonical hemodynamic response function than a simple curve fitting to unprocessed multiple echo measurements. We knew from previous research (Michálek et al., 2019) that exponential fitting amplifies noise, and also during previous research (Michálek, 2015), we had the experience that TV-based denoising removes spurious noise much more efficiently than other known methods. This is the reason why we chose 1D TV-minimization for fMRI time series denoising.

## 2 Methods

### 2.1 Data

Data used in this study were collected from nine healthy volunteers (five men, ranging from 25 to 39 years, mean age 29.8 years). The study was approved by the local ethics committee of Masaryk University, and all participants signed the informed consent. The measurement was performed at the Multimodal and Functional Imaging Laboratory at the CEITEC Masaryk University on a Siemens Prisma 3 T MR whole-body scanner with a 64-channel head–neck coil. The MRI protocol was identical for all participants and consisted of anatomical images and four fMRI runs. First, high-resolution *T_1_*-MPRAGE anatomical images were acquired for anatomical localization. In the second part of the protocol, we acquired four BOLD runs with two different acquisition settings. With each setting, one experimental task run and one resting-state run were recorded. The fMRI protocols were based on an MB-EPI BOLD sequence obtained from the Centre for Magnetic Resonance Research, University of Minnesota. For this article, only the first two runs of the four were used—one task and one resting-state run with identical acquisition settings (except the number of time frames) as follows: the field of view (FOV) was 192 × 192 mm, 48 transversal slices, voxel size 3 × 3 × 3 mm, and the three echo times TE = 15.00, 32.64, and 50.28 ms, respectively. The TE values were chosen according to the MR machine and sequence capabilities. The first TE was chosen approximately as the lowest possible value (rounded up to an integer), the second TE was as close as possible to the first one and was very similar to the typical optimal TE used in single-echo acquisition, and the third TE was as close as possible to the second one to not unnecessarily prolong the TR. The flip angle was 70°, based on the Ernst angle calculation. Because we used an averaged T_1_ time for gray matter from the literature and there can be subtle differences among the brain regions and individual subjects, we rounded it slightly down. We acquired the slices without a gap, i.e., the slice thickness of 3 mm is the final data resolution in the z-direction. The original data matrix size was 64×64×48 (in-plane resolution 64×64 pixels, 48 slices). The bandwidth was set to 2,230 Hz/pixel. We used the anterior–posterior phase encoding. The slices were set as transverse according to the AC-PC line. We used in-plane acceleration (GRAPPA) with a factor of 2 and a multiband acceleration factor of 2. Fat saturation was turned on. The repetition time TR was 1,800 ms. A total of 210 time frames (dynamic scans) were acquired during the *task* run (total acquisition time 06:36 min), and 312 time frames were obtained during the *resting-state* run (total acquisition time 09:40 min).

Figures 1–5, 7 and 9 in this report were created from the data of the first volunteer (sub-01), while Figures 6, 8 and 10–12 used data from all nine study participants.

**Figure 2:**
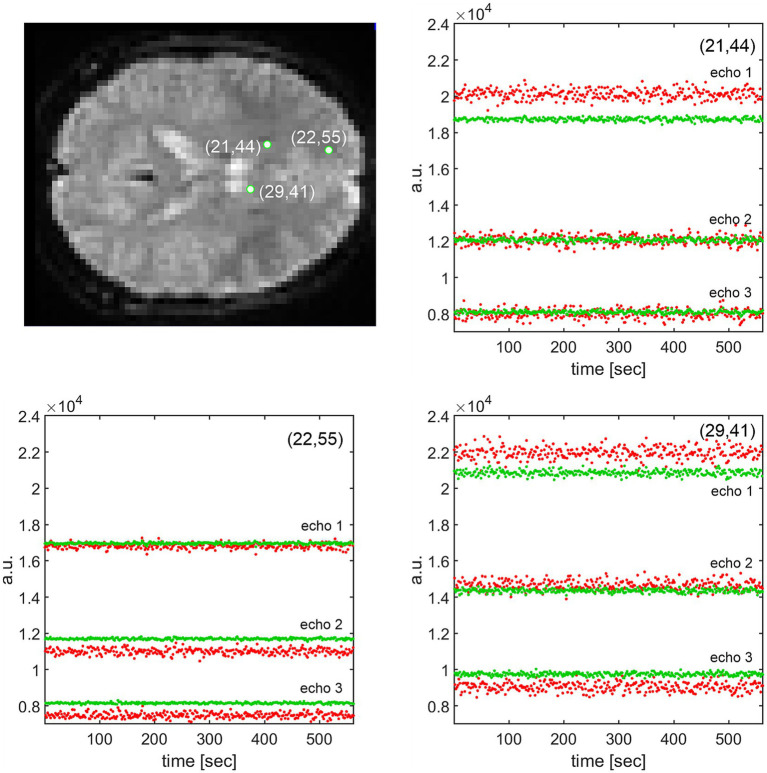
In red: raw (not denoised) time courses of three echoes at three randomly selected green-marked voxels from 312 realigned and MNI-coregistered fMRI frames (data from sub-01). In green: signal time courses at the same voxels after spatial Gaussian smoothing (FWHM = 5 voxels). At the voxel (21,44), e.g., Gaussian smoothing has squeezed the signal mean of the first echo by ~21.3% with respect to the raw signal, while the means of the second and third echoes remained unchanged. The means may contrarily increase in other voxels, such as (22,55) for echoes 2 and 3. As calculated in the text, the vertical drop of the Gaussian-smoothed first echo (21,44) has caused a massive distortion in the T_2_^*^ estimate, which increased by almost 88%.

**Figure 3:**
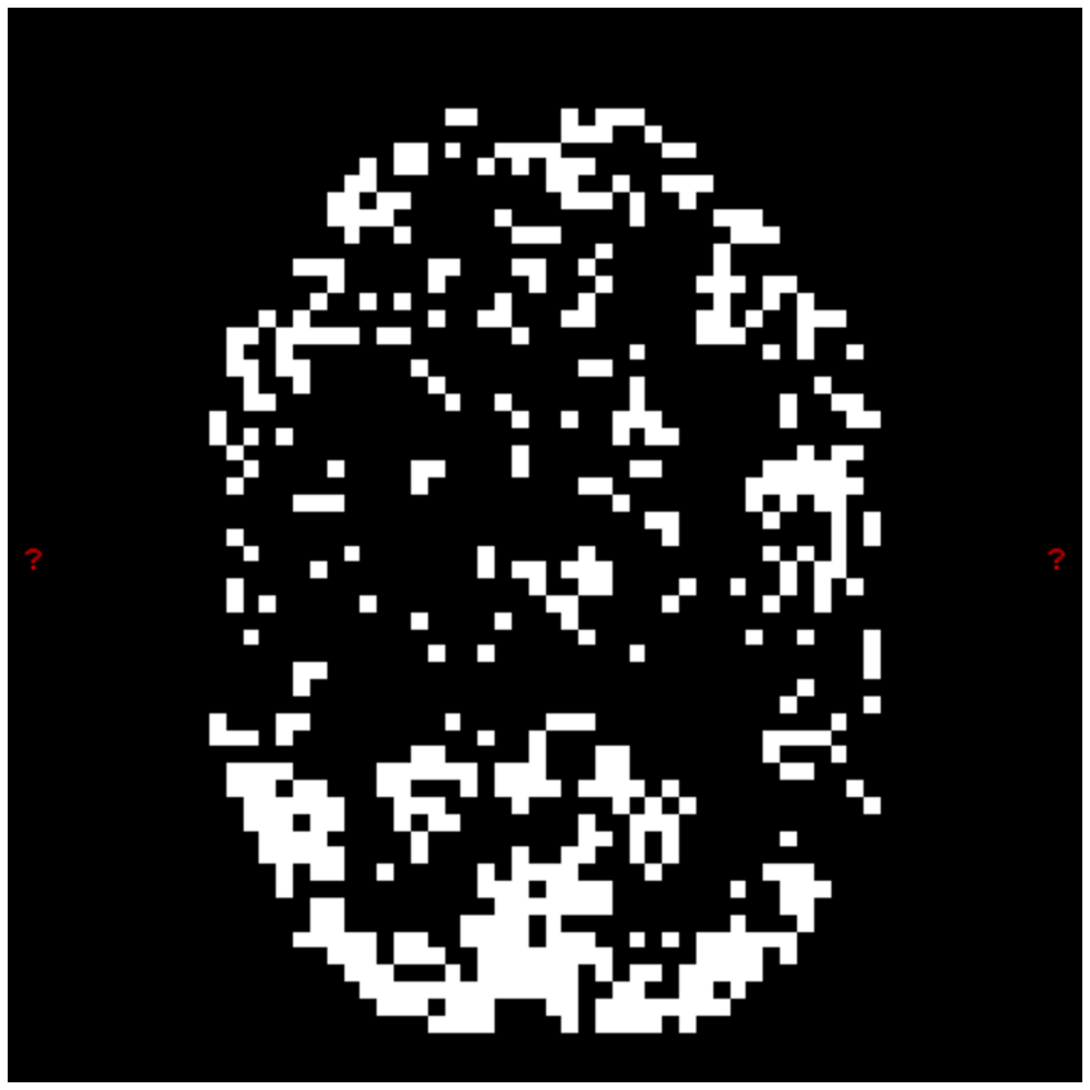
FDR-generated region of interest, middle slice. The highest density of the automatically computed ROI coincides with the visual cortex, as appropriate for the assessment of visual task fMRI data. The input to the above algorithm was the unprocessed echo 2 acquired from our sub-01 during the task described in 2.1.1. To get a fair comparison, the same ROI mask was used for CNR evaluation of T_2_^*^-TV fitting, optimal combination, *tedana*, and T_2_^*^-raw fitting.

**Figure 4:**
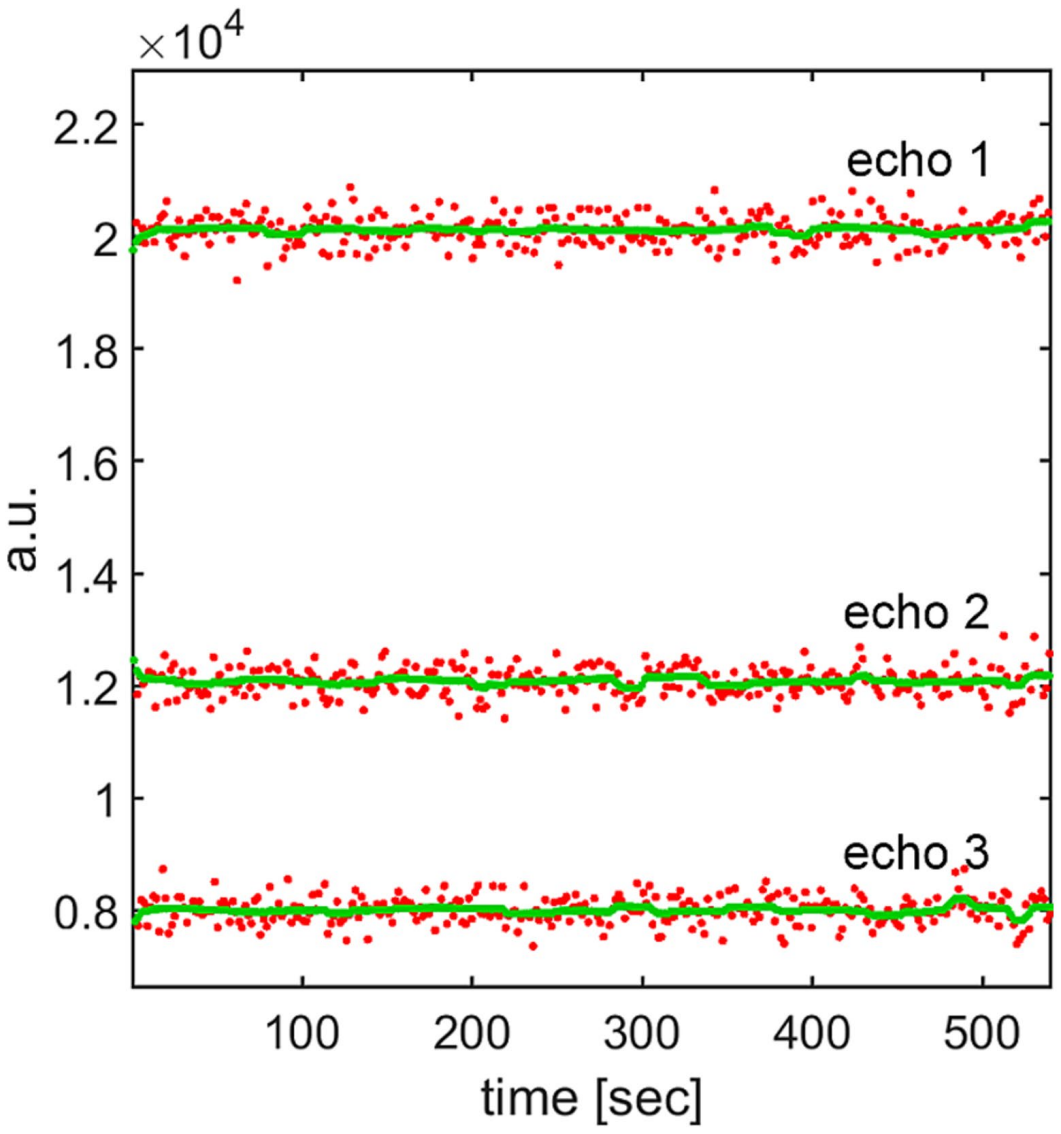
In red: raw time courses from 312 realigned and MNI-coregistered resting-state fMRI frames of three echoes picked at the same green-marked voxel (data from sub-01) as in Figure 1. The first echo has the highest mean. In green: the time courses of the signal at the same voxel after TV-based temporal restoration. All three TV-restored echoes exhibit substantially reduced noise while preserving local means, which will enable accurate dynamic T_2_^*^ estimation.

**Figure 5:**
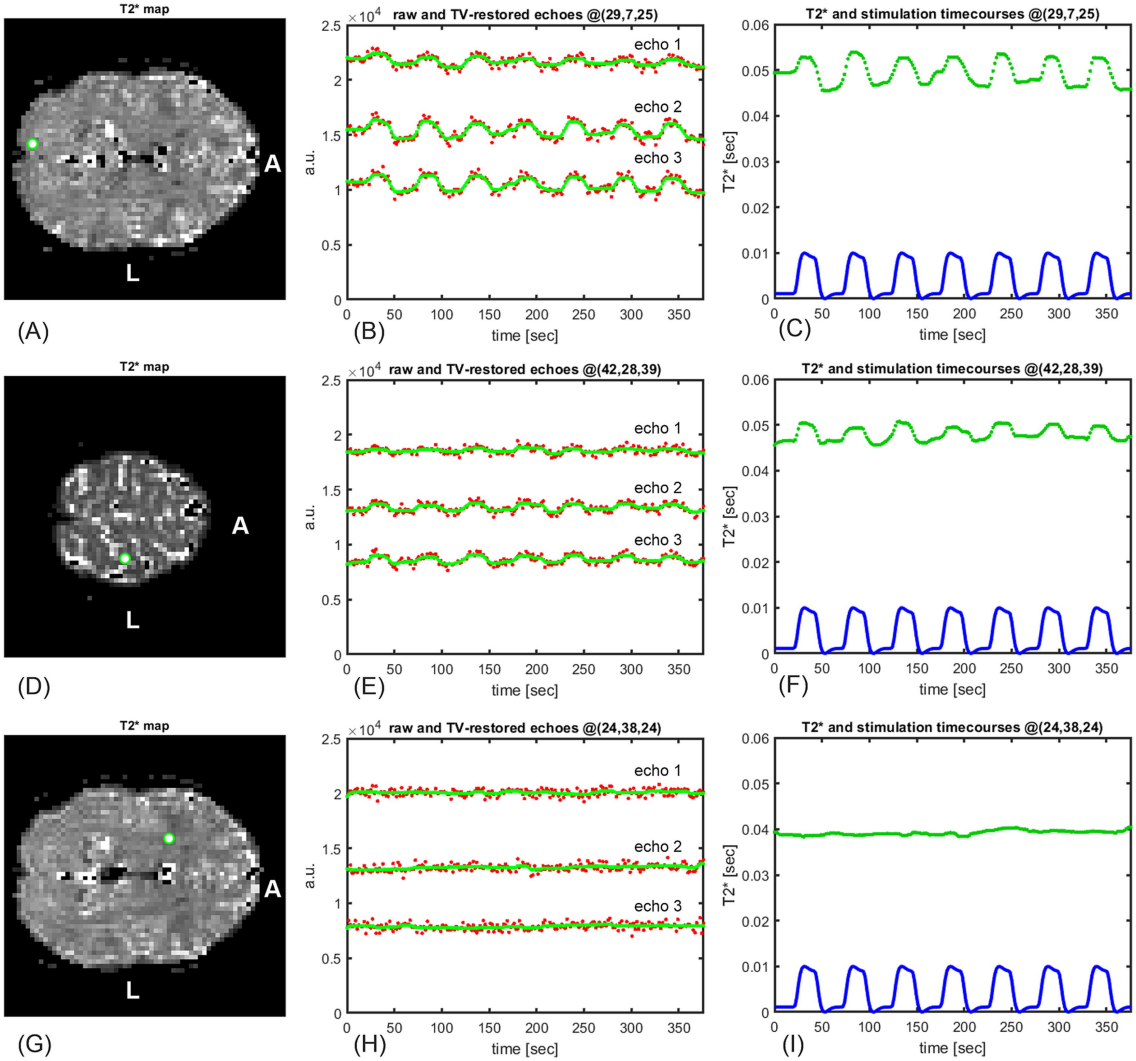
Voxelwise echo restoration and T_2_^*^ estimation in fMRI multi-echo series. **(A,D,G)** resulting T_2_^*^ map with a selection of sample voxels. **(B,E,H)** restored echoes (green) are—contrary to unrestored echoes (red)—smooth, as are experimental *in vivo* BOLD signal measurements. **(C,F,I)** owing to the smooth echoes, the estimated T_2_^*^ time courses (red) are also smooth. **(A,B,C)** the highest T_2_^*^ amplitudes were observed in the visual cortex, voxel (29, 7, 25). **(D,E,F)** the T_2_^*^ time course is synchronous with the theoretical hemodynamic response (blue) in a voxel from the motor cortex (42,28,39) of the brain. **(G,H,I)** voxel (24,38,24) belongs to the transition between gray and white matter; hence, the particular part of the brain should not be activated by the task. This is confirmed by **(I)**, where—unlike in **(C,F)**—no similarity between the theoretical hemodynamic response and the T_2_^*^ time course is noticeable.

**Figure 6:**
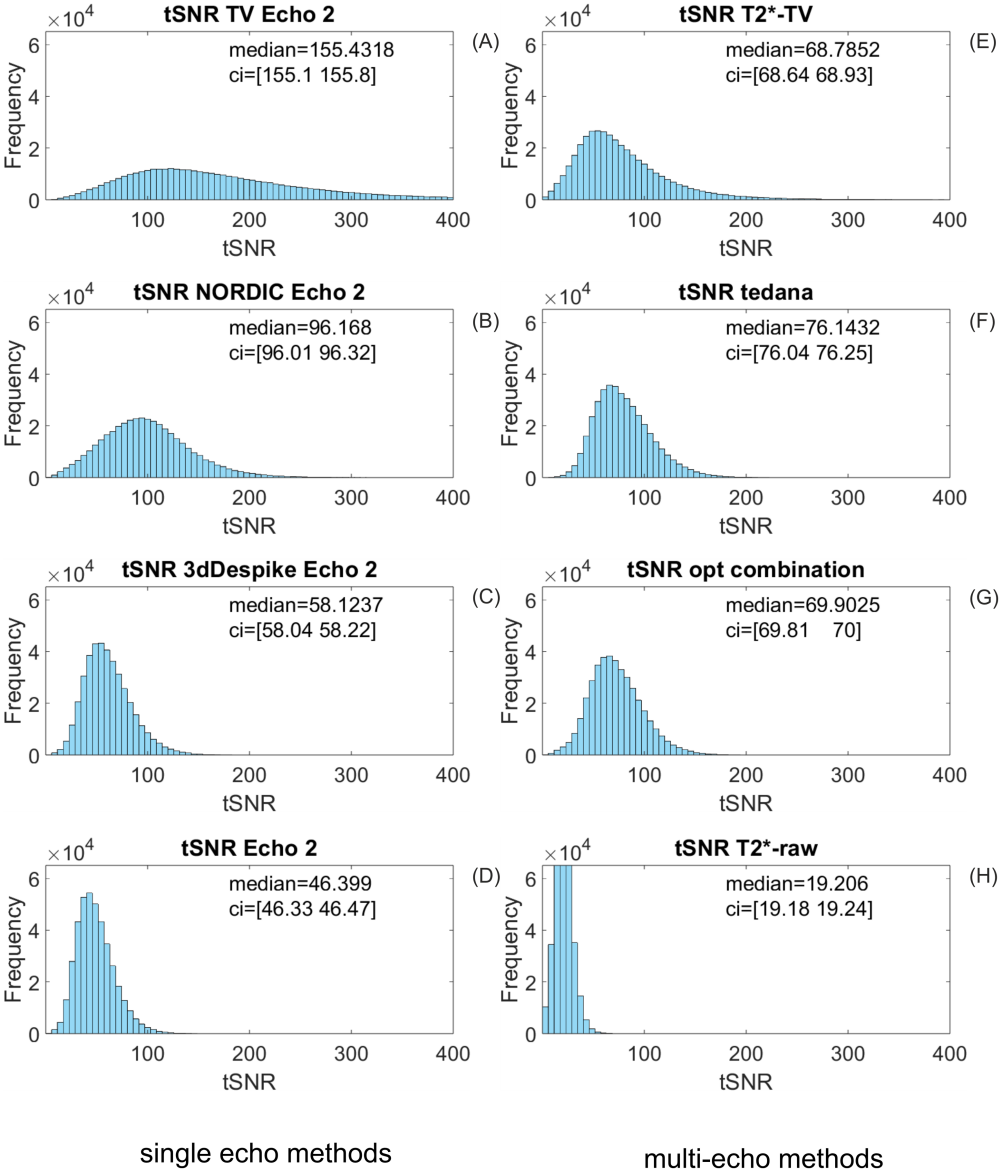
Comparison of tSNR distributions for eight different signal preprocessing variants of all *resting-state* BOLD signal time courses over the nine study participants. Depending on whether researchers consider single-echo or multi-echo fMRI data, the left or the right column may be more relevant. tSNR medians and their respective confidence intervals are included with each plot. Left column: **(A)** *TV-l2* denoising of echo 2 produces the highest tSNR of the four single-echo BOLD signal processing methods. **(B)** The single-echo NORDIC-denoised time course features better tSNR distribution than **(C)** the 3D-despiked echo. The 3D-despiked echo 2, in turn, has a somewhat better signal-to-noise ratio than **(D)** the raw echo 2, which is often used for fMRI data analysis. Right column: multi-echo preprocessing of fMRI data does not necessarily lead to higher tSNR values. The *tedana* package **(F)** has the highest tSNR of the multi-echo methods, yet it is outperformed by the single-echo methods **(A,B)**. tSNR of the T_2_^*^ mapping from total-variation-denoised three echoes **(E)**, is, for resting-state data, about on a par with the weighted sum of three echoes, referred to as “optimal combination,” **(G)**. An important difference, however, is that T_2_^*^ mapping delivers quantitative output measured in time units (seconds), while the output of *tedana* or optimal combination is in arbitrary units. The “optimal combination,” essentially a weighted sum of three echoes, acts as a low-pass filter, and its tSNR is better than that of the plain unprocessed echo 2 **(H)**.

**Figure 7:**
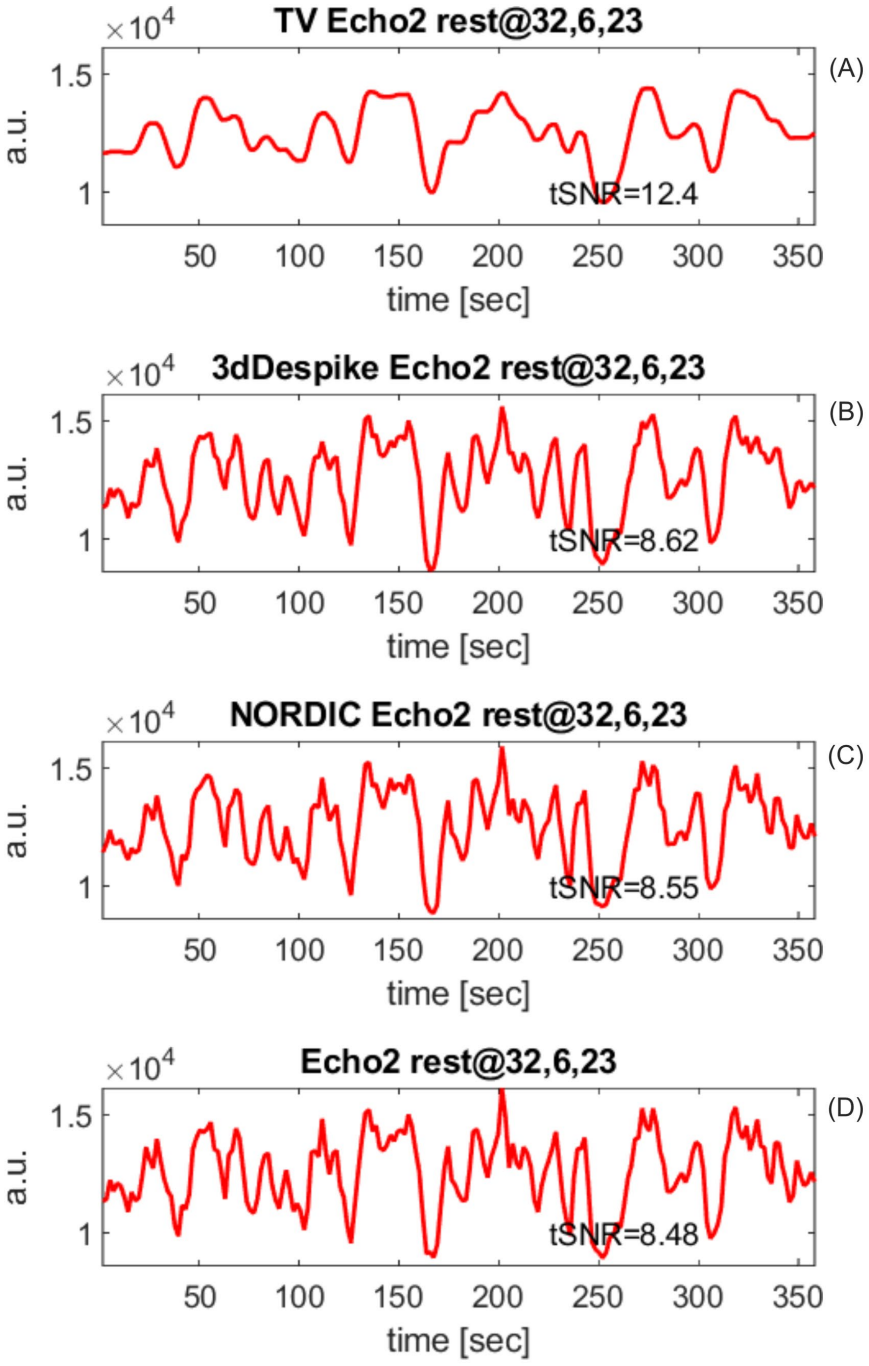
Participant sub-01, voxel (32,6,23). **(A)** The total-variation-denoised resting-state echo 2 has the highest tSNR and is—contrary to the other time series—smooth. **(B)** Second-best tSNR was generated by AFNI’s *3dDespike* function. **(C)** *NORDIC*-denoised echo 2. **(D)** tSNR of unprocessed echo2. For the participant sub-01, tSNRs of **(B,C,D)** were not much different.

**Figure 8:**
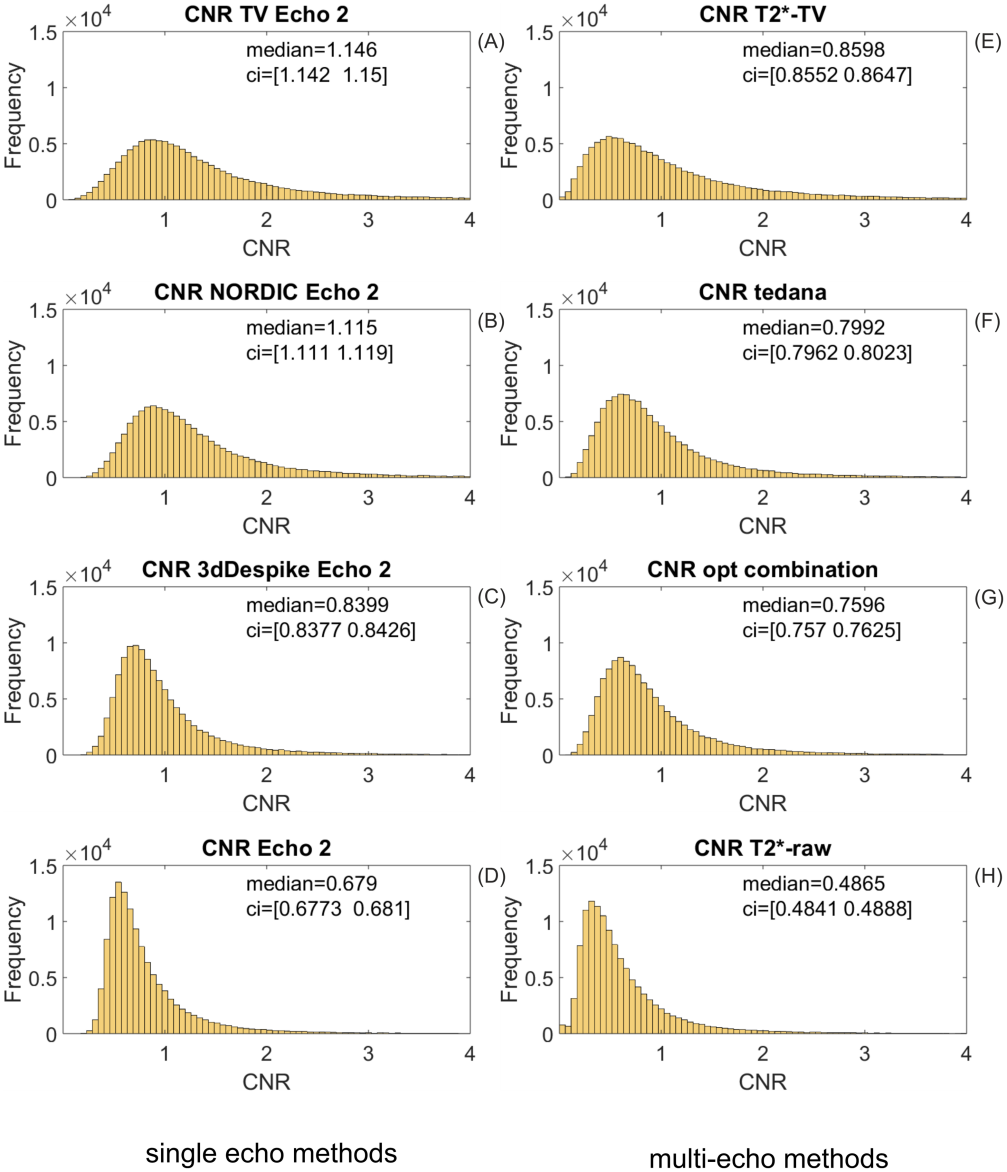
Comparison of CNR distributions over all *task* runs of nine study participants for four types of single-echo task-based BOLD signal processing and four types of multi-echo task-based BOLD signal processing. **(A)** The *TV-l2* denoising of echo 2 produces the highest CNR of the four single-echo BOLD signal processing methods. **(B)** The CNR distribution achieved by single-echo NORDIC denoising is higher than that in **(C)**, where the CNR histogram for the 3D-despiked echo is shown. The 3D-despiked echo 2 has a better contrast-to-noise ratio than the raw echo 2 **(D)**, which is frequently used for fMRI data analysis. **(E)** T_2_^*^ mapping from three TV-denoised echoes. T_2_^*^-TV achieves the highest CNR values of multi-echo methods. **(F)** The *tedana.py* implementation of the ME-ICA package by Kundu et al. (2012) yields the second highest CNR in the task-based data comparison. **(G)** The CNR distribution of the “optimal combination” that was computed using the script *t2smap.py* lies only slightly below that of *tedana*, script *tedana.py*. **(H)** T_2_^*^ mapping from raw echoes amplifies noise, thus deteriorating the contrast-to-noise ratio.

**Figure 9:**
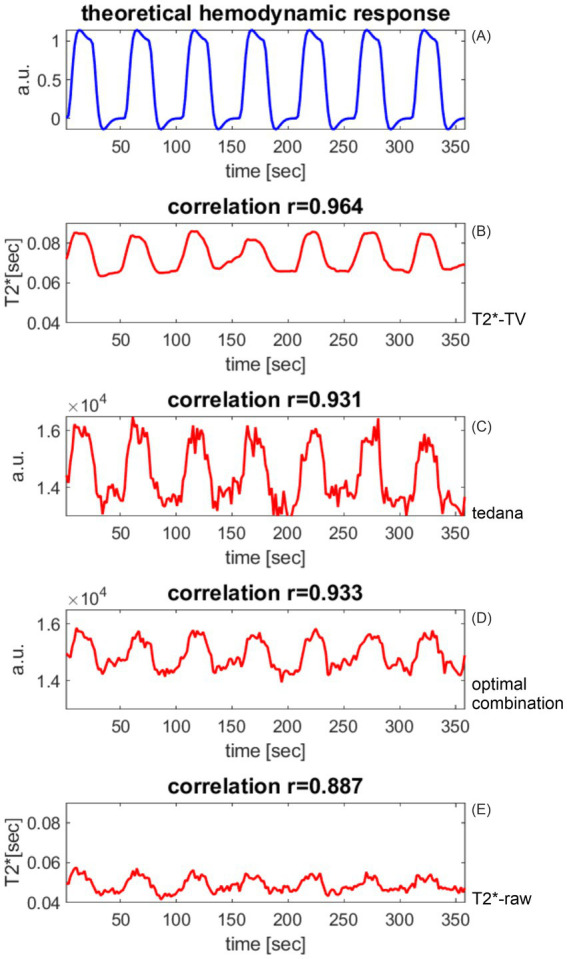
Similarity between the shape of the theoretical hemodynamic response and the BOLD signal calculated from three echoes at the best-matching voxel. **(A)** Theoretical hemodynamic response. **(B)** T_2_^*^ mapping from three TV-denoised echoes. **(C)** *tedana.py*-denoised BOLD signal; **(D)** “optimal combination” of three echoes: weighted sum of the three echoes contributes to denoising. **(E)** T_2_^*^ estimate from three unprocessed echoes: the noise in the measured three echoes is amplified by the T_2_^*^ mapping, and results in low correlation values. The TV-based T_2_^*^ signal **(B)** is smooth, as required by the theory of BOLD response to stimulation and confirmed by invasive BOLD signal measurements. Smoothness was achieved thanks to the denoising properties of the total-variation-based Rudin–Osher–Fatemi algorithm, which—unlike classical approaches—suppresses *temporally isolated local disturbances* of limited extent rather than *additive time series* (fourier components, principal components, independent components, etc.).

**Figure 10:**
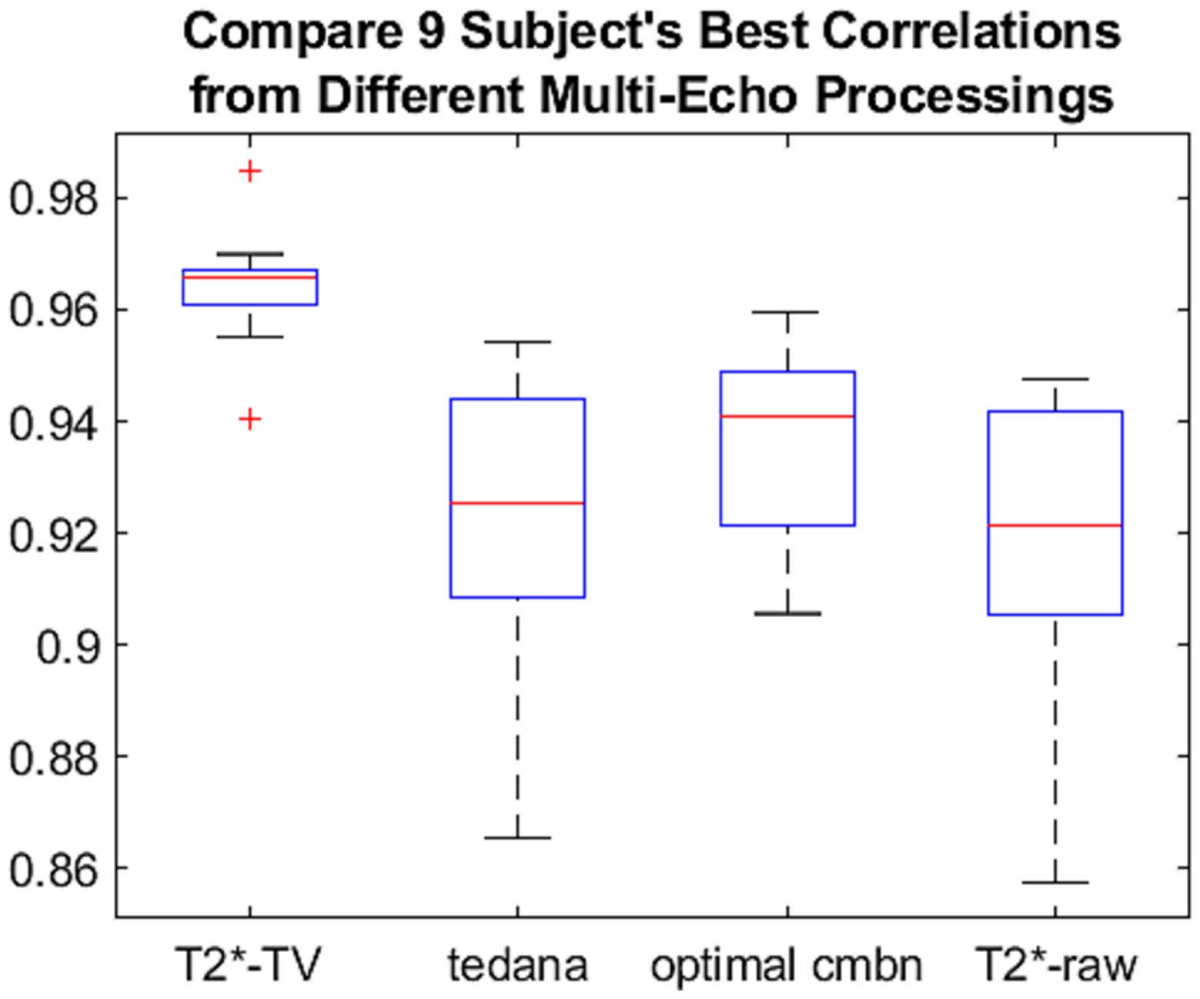
Boxplots of the medians (red lines) and interquartile ranges (box heights) for nine subjects of the best correlation values between fMRI signals resulting from different types of multi-echo processing and the normalized hemodynamic response. As explained in Table 1, the median for the T_2_^*^-TV method (red line) is significantly higher than correlation medians obtained for the other multi-echo BOLD signal processing methods. The narrow IQR of the T_2_^*^-TV indicates that the correlation values are less dispersed for the T_2_^*^-TV method than for other types of multi-echo fMRI signal processing.

**Figure 11:**
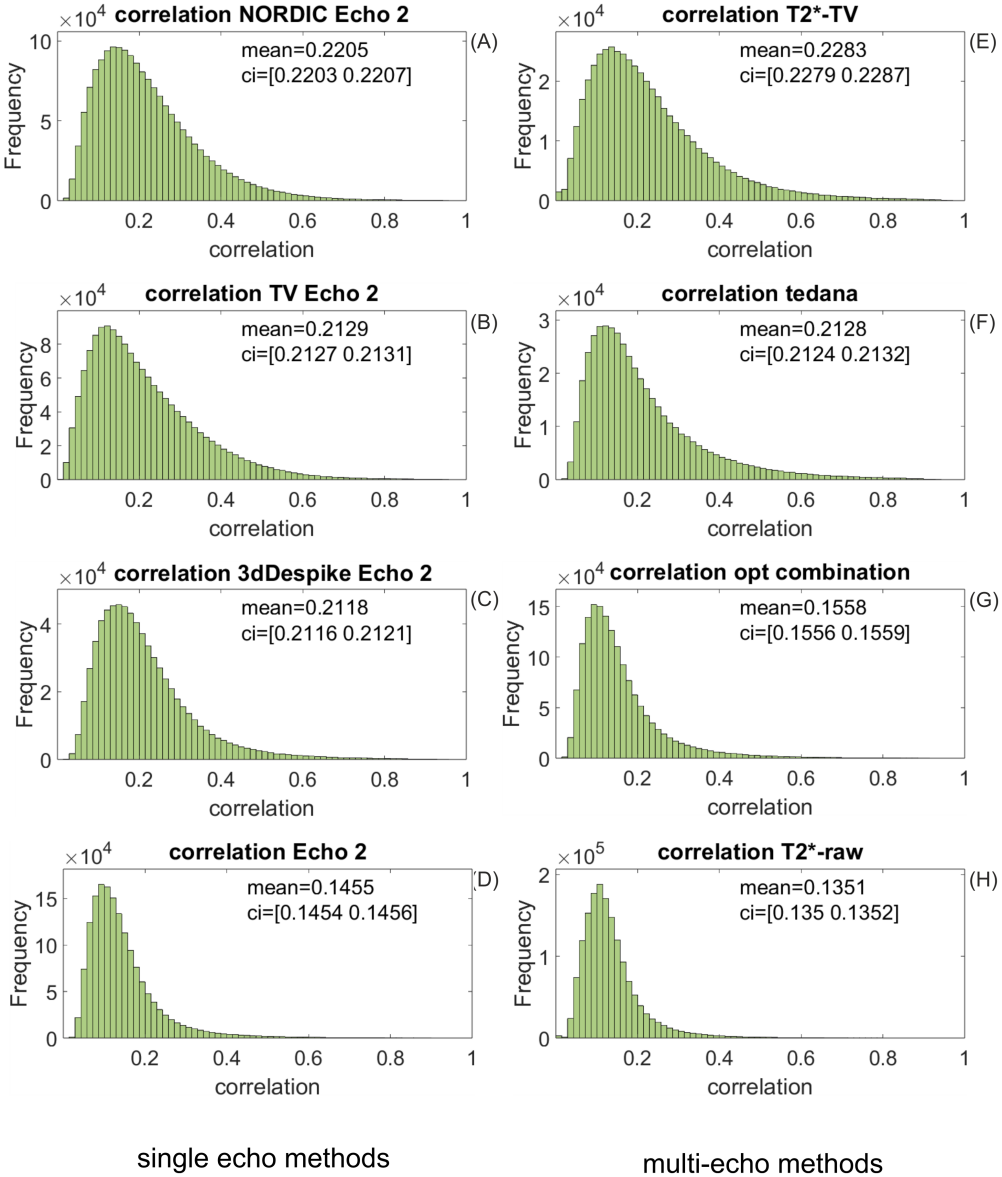
Histograms of the mean normalized correlation between the normalized theoretical hemodynamic response to stimulation and the normalized outcome of four single-echo and four multi-echo fMRI preprocessing methods. **(A)**. Among single-echo methods, the NORDIC denoising yields the highest stimulus–response correlation, with the distribution for TV-based echo denoising **(B)** visually almost indistinguishable. All three single-echo denoising methods, including 3dDespike **(C)**, improve correlation substantially compared to **(D)**, the unprocessed echo 2. Total-variation-based T_2_^*^ mapping **(E)** yields the best correlation among the multi-echo methods, with *tedana* **(F)** closely following. For the “optimal combination” **(G)**, the effect size is surprisingly low—the mean value is barely higher than that of the unprocessed single-echo signal **(D)**. T_2_^*^ mapping from raw echoes **(H)** deteriorates the correlation, probably due to noise amplification through the log-linear fitting.

**Figure 12:**
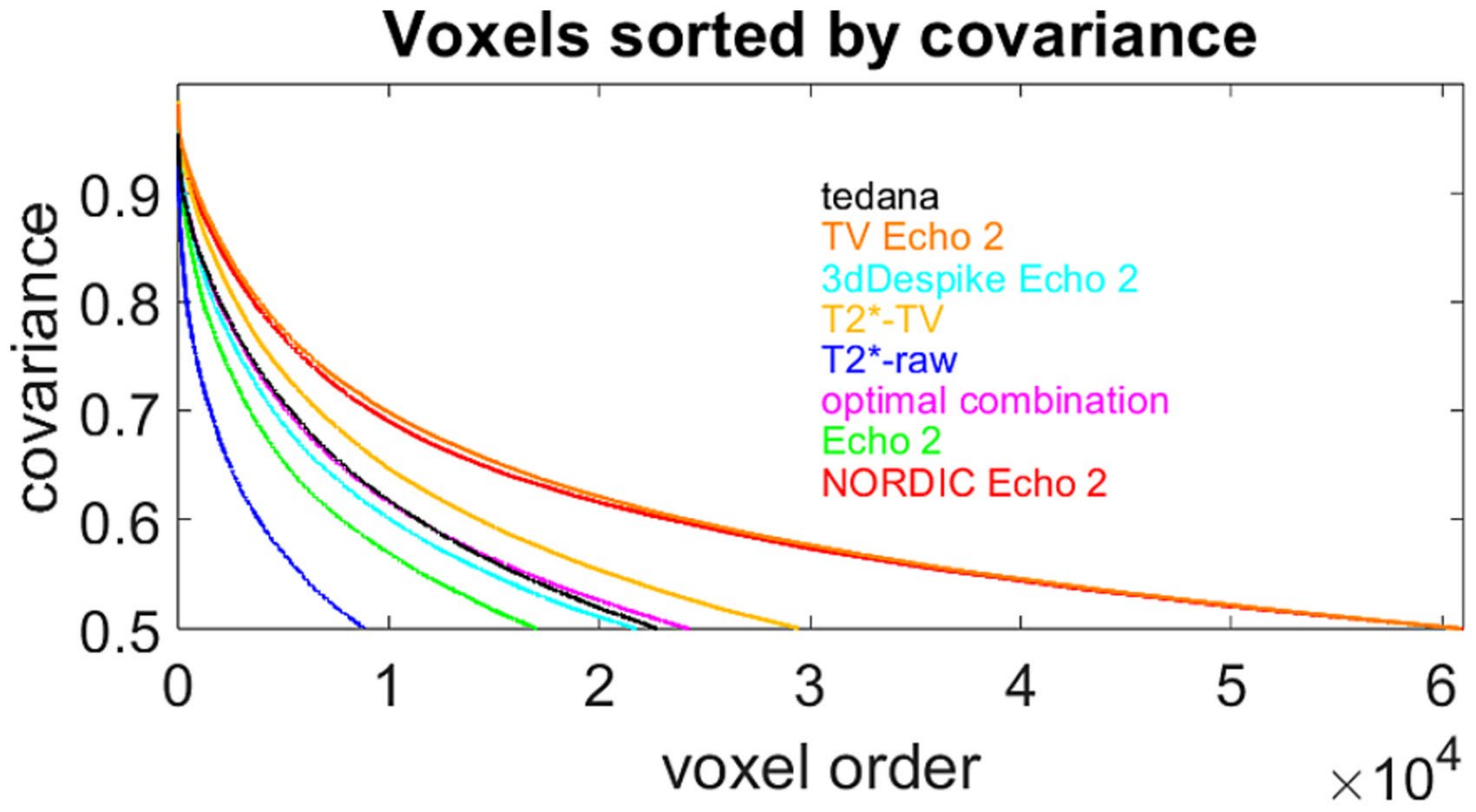
Another view of the effect sizes. The colored lines display, for all eight fMRI preprocessing methods, sorted voxels whose correlation with the theoretical response to stimulation is greater than 0.5. The intersection of each curve with the horizontal axis suggests how many such voxels were produced by the respective method. Single-echo NORDIC denoising and the novel TV-based echo denoising produced the highest numbers of high-correlation voxels, as expected from the good agreement between the histograms in Figures 12A,B.

#### 2.1.1 fMRI task

The fMRI block-design task consisted of two regularly alternating epochs. The first epoch was the baseline, with the volunteer instructed to lie still and fix their eyes on a red cross on a black background in the middle of the stimulation screen. In addition, the subject was advised not to think intensely. This epoch lasted 30.025 s. A second epoch followed in which the red numbers 1, 2, 3, and 4 in a series of 10 gradually appeared on a checkerboard background, and the subject was asked to press simultaneously the corresponding buttons. This active measurement period lasted 21.35 s. The sequence of the two epochs was repeated seven times within one fMRI run.

#### 2.1.2 Preprocessing of the multi-echo datasets

Preprocessing was done using the SPM12 software (available from https://www.fil.ion.ucl.ac.uk/spm/software/download/) and in-house scripts running under MATLAB 2017b. To allow testing of the T_2_^*^ estimation on data that underwent various degrees of preprocessing, the fMRI time series for each of the three echoes was processed individually in the following stages:

- Correction of movement by the “realign and unwarp” SPM12 function.
- Spatial normalization into the MNI space, with the same warp applied to all echoes.
- Regression of white matter (WM) and cerebrospinal fluid (CSF) representative signals as predictors of artificial (non-neural) fluctuations. We used a simple method based on a general linear model (GLM) with three columns to regress out the main WM and CSF effects. Two columns represented the signal fluctuation in WM and CSF, respectively, and the third column was used to fit the constant term. The fitted effect of WM and CSF was subtracted from the data. The effect of the constant term was not subtracted to avoid a change in the mean signal intensity. The representative time series of WM and CSF were constructed as the first principal components of the set of detrended time series that were delimited by an *a priori* mask based on tissue probability maps from SPM12 for WM and CSF, respectively.
- Spatial smoothing with a Gaussian filter of FWHM (full width at half maximum) = 5 × 5 × 5 mm^3^.

#### 2.1.3 Dimensions of the multi-echo datasets

The acquired multi-echo fMRI data were accommodated in five-dimensional (5D) datasets of the shape [*R* × *C* × *S* × *E* × *F*] with:

*R*: number of rows in the 3D image grid.

*C*: number of columns in the 3D image grid.

*S*: number of slices in the 3D image grid.

*E*: number of echoes captured during one repetition time.

*F*: number of acquired frames.

The symbol *Y* denotes the 5D fMRI dataset:


$$Y=(\varphi_{r,c,s,e,v})\in R^{R\times C\times S\times E\times F}$$

where $\left(\varphi_{r,c,s,e,v}\right)$ denotes a five-dimensional real matrix from $R^{R\times C\times S\times E\times F}$ , the space of all real-valued matrices with dimensions [*R* × *C* × *S* × *E* × *F*]. In this notation, $\varphi_{r,c,s,e,v}$ is the measured BOLD signal value of the echo e at the space location r,c,s , and volume v . The five-dimensional matrix Y is the data object we work with when processing a multi-echo fMRI dataset in MATLAB.

The number of rows *R* = 64, number of columns *C* = 64, number of slices *S* = 48, and number of echoes *E* = 3 were common to the resting-state and the task datasets. The resting-state data comprised *F* = 312 time frames, and the task data comprised *F* = 210 frames.

### 2.2 Pitfalls of T_2_^*^ mapping from three echoes

For the transverse magnetization decay, we assumed the following monoexponential model:


$$e=S_{0}\exp(-t/T_{2}^{*})$$

where *t* is the echo time, *S_0_* is the initial signal intensity, T_2_^*^ is the “observed” or “effective” decay time constant, and *e* is the echo signal intensity. With two unknowns *S_0_* and T_2_^*^, T_2_^*^ can be estimated by fitting an exponential to the echoes if at least two echoes measured during one repetition are available.

#### 2.2.1 Gaussian spatial smoothing changes the mean values of the echo time courses

Preprocessing of the fMRI signal is, to a large extent, standardized and involves correction steps for random confounding signals (“noise”) such as head movement, magnetic field inhomogeneity, or slice timing differences. In widely used program packages for fMRI analysis, such as SPM12 (https://www.fil.ion.ucl.ac.uk/spm/software/download/), corrections usually involve the following preprocessing steps:

- Motion correction and unwarping.
- Spatial normalization, including co-registration.
- Spatial smoothing of the acquired frames.

For spatial smoothing, a Gaussian low-pass filter is used in SPM12. We parametrized the Gaussian filter by choosing FWHM = 5 mm in all directions. Using this parametrization, it turned out that in most voxels, spatial smoothing vertically shifted the mean values of the BOLD echo time series, as illustrated in Figure 2. The time course (312 scans in total) of the Gaussian-smoothed echo 1 (green) at the voxel (21,44) lies approximately 21% below the unsmoothed BOLD signal mean (red). The shift resulted from the Gaussian smoothing, which calculates a voxel’s value by weighted averaging in three directions of the image rows, columns, and slices. The averaging shifts a voxel’s time series mean even if the voxel lies on a sharp boundary between different tissue types, beyond which the voxel’s value should not propagate. As for the echoes 2 and 3, Gaussian smoothing left the signal means more or less unchanged. By way of example, at voxels (22,55) and (29,41), the echo time series are shifted quite differently.

Vertical shifts of the Gaussian-smoothed echo time series can be explained as follows:

An SPM MATLAB code inspection (spm_smooth.m, spm_smoothkern.m) shows that the 3D Gaussian filter with FWHM = 5 mm has, at voxel distances [−6, −3, 0, 3, 6] mm from the central voxel, the following relative weights (in %): [1.9835, 37.5354, 100.0000, 37.5354, 1.9835]. Voxels at distances larger than 6 mm from the center have weights less than 1% of the central voxel, and we neglect them.

First, a simplified 1D numerical example: if we assume that all five voxels [−6, −3, 0, 3, 6] on the 1D filter grid are white (have value 1), then the Gaussian-smoothed central voxel will again be white, and analogously for all five voxels black (value 0). If, however, the voxel 0 lies on the boundary between white and black, e.g., [−6, −3] are black and [0, 3, 6] are white, then the Gaussian-smoothed voxel 0 will have the brightness value of only 0.7793 rather than 1, i.e., 22% lower.

This is only a 1D example. In the real 3D fMRI volume, the Gaussian filter works with 5 × 5 × 5 = 125 neighboring voxels that have relative weights >1%. In 3D, percentual brightness shifts may be larger than in the 1D case, depending on how many voxels in the 5 × 5 × 5 voxel cube possess brightness that is significantly different from the central voxel being smoothed.

We work with three MRI magnitude images that were acquired at the time instants $TE_{1}=15.00,TE_{2}=32.64,TE_{3}=50.28$

If, for the first echo, sufficiently many unsmoothed voxels on the 5 × 5 × 5 voxel grid are brighter (have higher initial signal intensity *S_0_*) than the voxel being smoothed (central voxel), then the mean level of echo 1 time series at that voxel will be shifted upwards, and vice versa.

Vertical shifts in the second and third echo time series may be caused by the fact that within the 5 × 5 × 5 voxel surroundings captured by the Gaussian, there are tissues with different relaxation rates R_2_^*^. Tissues with faster relaxation (higher R_2_^*^) fade out sooner than those with lower R_2_^*^. Depending on the Gaussian weighting of fast-relaxation and slow-relaxation voxels within the 5 × 5 × 5 voxel volume, this may result in a vertical shift of the time series mean of echoes 2 and 3 or may leave the mean unchanged. As the shift in the first echo time series depends on the initial signal intensity, while the second and third time series shift depend on the voxel relaxation rate R_2_^*^ (or time T_2_^*^), the time series shifts of echoes 1, 2, and 3 will, in general, differ. Empirically, this is confirmed by the three echo triples in Figure 2.

#### 2.2.2 Changes in the mean value of the echo time courses distort the estimates of T_2_^*^ values

Obviously, T_2_^*^ estimated from the green, spatially smoothed, vertically shifted BOLD echo time series in Figure 2 will be different from the T_2_^*^ calculated from instantaneous means of the red, unfiltered echoes: if we assume the monoexponential magnetization decay model $e=S_{0}\exp(-t/{T_{2}}^{*})$ with initial signal intensity *S_0_*, then, for two echoes *e*_1_ and *e*_2_ acquired at times *t*_1_ and *t*_2_ after the RF excitation, T_2_^*^ can be estimated using the log-linear fit $T_{2}^{*}\approx (t_{2}-t_{1})/(\log e_{1}-\log e_{2})$ . If the time difference between the two echoes is $(t_{2}-t_{1})=17.64$ ms and the unsmoothed first two echo values are as in Figure 2, voxel (21,44), (red dots) $e_{1}\dot{=}20200$ and $e_{2}\dot{=}12100$ , respectively, then we get an estimate $T_{2}^{*}=34.42\;\mathrm{ms}$ . If we instead estimate T_2_^*^ from the spatially Gaussian-smoothed time courses (green dots) with $e_{1}\dot{=}15900$ and $e_{2}\dot{=}12100$ , we get the distorted estimate $T_{2}^{*}=64.59\;\mathrm{ms}$ . This means that a −21% distortion of the first echo by Gaussian smoothing caused an almost 88% distortion of the T_2_^*^ estimate and made it unusable. Clearly, distorted T_2_^*^ estimates from Gaussian-smoothed echoes no longer properly reflect the blood oxygenation level. This justifies the following requirement:

Requirement. T2^*^ estimation must be done from multi-echo BOLD time series *not* processed by Gaussian spatial smoothing because spatial smoothing destroys echo mean values and, consequently, the T_2_^*^ estimates.

Authors of the online manual: https://tedana.readthedocs.io/en/stable/multi-echo.html (tedana, n.d.) also recommend doing any step that will alter the relationship of signal magnitudes between echoes, such as spatial smoothing, after denoising the echoes. However, they do not show how the amplitudes may change due to Gaussian spatial smoothing, nor do they explain why the signal amplitudes are being altered. We have not found an explanation in their recommended reference, DuPre et al. (2021), either.

### 2.3 Restored BOLD signal should be smooth and preserve the measured local mean values of the echoes

#### 2.3.1 *In vivo* measurements of the BOLD echoes indicate that the physiological BOLD signal is smooth

In the past, the biophysical and physiological sources of BOLD fMRI signals were analyzed by different researchers in animal studies, which afforded the systematic assessment of physiological sources and the combination of fMRI with invasive approaches. Silva et al. (2000) investigated BOLD changes elicited by somatosensory stimulation of the forepaw in rats, and Nagaoka et al. (2005) examined BOLD response to visual stimulation in the primary visual cortex of cats. Their results were reviewed by Kim and Ogawa (2012). The measured time courses of BOLD responses to a forepaw stimulus in the rat somatosensory cortex in Kim and Ogawa (2012), Figure 5A and to a visual stimulus in the cat visual cortex in Kim and Ogawa (2012), Figure 5B suggest that, after averaging out the measurement noise, the BOLD signal is *smooth*.

However, in our GRE-measured BOLD echoes, all three echoes fluctuated randomly without showing any regular pattern (Figure 1) and did not have the appearance of a sampled smooth signal.

#### 2.3.2 Formulation of temporal BOLD signal restoration as minimization of total signal variation

In a first attempt to remove noise while preserving the signal mean values for reasons discussed in Section 2.2.2, we applied *temporal* Gaussian filtering separately to each of the three echoes at every voxel of the brain. However, as the Gaussian is a low-pass filter, there still remained large oscillations in the filtered signal at voxels with fast fluctuations in the unprocessed data because low-pass filtering does not remove fast signal changes.

To make denoising frequency independent, we propose to specify the temporal BOLD signal restoration task in terms of the following two requirements:

- As the *physiological* BOLD signal measured in animal experiments can be assumed to be *smooth*, minimize the variation of the *measured* BOLD signal to make the *restored* time course smooth (*smoothness*).
- At the same time, keep the restored signal values as close to the local signal mean (i.e., mean signal value in the neighborhood of the current timepoint) as possible (*data fidelity*).

The trade-off between the smoothness and the data fidelity is controlled by a weight *μ*. Mathematically, this can be formulated as a one-dimensional (1D) *TV*–*l_2_* (total variation and *l_2_* distance) optimization problem, whose two-dimensional version is widely known in the image processing literature as the Rudin–Osher–Fatemi (ROF) model for image denoising (Rudin et al., 1992).

The solution to the *TV*–*l_2_* problem is known to have favorable properties: contrary to linear filters (e.g., low-pass such as Gaussian, high-pass, etc.) that suppress nuisance *signals* depending on their *frequency*, in the nonlinear *TV*–*l_2_* filtering disturbing *events* are removed depending on their *value* and *extent* along the timeline: it is shown in Chambolle and Pock (2016) that gradually decreasing the data fidelity weight *μ* results in removing nuisance structures with increased *size* and *contrast*.

To enforce smoothness of the T_2_^*^-time courses, each of the three echoes of the 5D dataset must be restored separately voxel by voxel. For notational simplicity, the symbols used below in the derivation of the algorithm may refer to any voxel of any of the echoes e=1,e=2,ore=3 .

We will denote with $\vec{b}$ the measured 1D time series of the fMRI values of the echo *e* at some voxel (r,c,s) :


r∈{1,2,…,R},c∈{1,2,…,C},s∈{1,2,…,S},e∈{1,2,3}

$$\vec{b}=Y(r,c,s,e)$$

Similarly, we will denote with $\vec{u}$ the estimate of the restored, noise-free, time course at (r,c,s,e) , and with ${\vec{u}}_{k}$ the estimate in the *k*-th iteration.

#### 2.3.3 Objective function

The solution to the BOLD time course restoration, formulated as the *TV*–*l_2_* problem, requires that the sum:


$$G(\vec{u})=\mathrm{TV}(\vec{u})+\frac{\mu }{2}{\left\|\vec{u}-\vec{b}\right\|}_{2}^{2}$$

be minimized. Here, $\mathrm{TV}(\vec{u})$ denotes the TV of the restored temporal signal and is defined as the sum of magnitudes of the forward differences of the denoised signal $\vec{u}$ :


$$\mathrm{TV}(\vec{u})=\sum_{v=1}^{F}|\nabla \vec{u}(v)|=\sum_{v=1}^{F}|{\vec{d}}_{v}\cdot \overset{\to |}{u}={\left\|D\vec{u}\right\|}_{1}$$

and ${\left\|\vec{u}-\vec{b}\right\|}_{2}^{2}$ is the squared error between the measured echo $\vec{b}$ and its estimate $\vec{u}$ .

The symbols have the following meanings:

*F*: number of measured fMRI frames.

*v*: frame index.

$\vec{u}$ : restored 1D time course of the BOLD signal at some voxel.

$\nabla \vec{u}$ : forward differences between neighboring timepoints.

${\vec{d}}_{v}$ : *v*-th vector whose dot product with $\vec{u}$ yields the *v*-th difference $\nabla \vec{u}(v)$

*D*: [*F* × *F*]-matrix whose *v*-th row is ${\vec{d}}_{v}^{T}$

With the above notation, the objective function to be minimized for 1D BOLD signal restoration can be written as follows:


$$G(\vec{u})={\left\|D\vec{u}\right\|}_{1}+\frac{\mu }{2}{\left\|\vec{u}-\vec{b}\right\|}_{2}^{2}$$

The objective function is, up to a projection matrix *R*, formally equal to that of Michálek (2015, p. 6). Therefore, it can be minimized using the inexact alternating direction method of multipliers (ADMM) algorithm derived there, with the projection matrix *R* replaced with the identity matrix *I*.

#### 2.3.4 Partially inexact ADMM for denoising one-dimensional time series

Eckstein and Yao (2017) proved the convergence of two new approximate versions of the ADMM. Each of the methods permits both subproblems to be solved inexactly. The first method uses an absolutely summable error criterion. The second method uses a relative-error criterion and an auxiliary iterate sequence that enables relative-error approximate implementation of augmented Lagrangian algorithms.

To cast the iterations of the inexact ADMM in Michálek (2015) into the flow of Algorithm 2 in Eckstein and Yao (2017), only the relative-error criterion and the auxiliary iterate sequence need to be added. The complete algorithm for 1D BOLD signal restoration then assumes the following form:

##### 2.3.4.1 Algorithm

Partially inexact ADMM for 1D denoising with a relative error criterion.

**Initialization:** pick scalar parameters μ>0,β>0 and σ∈(0,1] and initial points ${\vec{u}}^{0},{\vec{r}}^{0},{\vec{\lambda }}^{0},{\vec{w}}^{0}\in R^{F}$

**for** *k*=0, 1, 2,.. **do**

${\vec{u}}^{k,0}={\vec{u}}^{k}$

**repeat** {**for** *l=*1,2,..}

$\vec{g}=\beta \cdot D^{T}(D{\vec{u}}^{k,l-1}-{\vec{w}}^{k}-\frac{{\vec{\lambda }}^{k}}{\beta })+\mu ({\vec{u}}^{k,l-1}-\vec{b})$

$\alpha_{k}=\frac{{\vec{g}}_{k}^{T}{\vec{g}}_{k}}{{\vec{g}}_{k}^{T}A{\vec{g}}_{k}}$ , where $A=\beta \cdot \sum_{i}D_{i}^{T}D_{i}+\mathrm{\mu I}$ ,

${\vec{u}}^{k,l}={\vec{u}}^{k,l-1}-\alpha \vec{g}$

**until** $\frac{2}{\beta }|\langle {\vec{r}}^{k}-{\vec{u}}^{k,l},\vec{g}\rangle |+{\left\|\vec{g}\right\|}^{2}\leq \sigma {\left\|D{\vec{u}}^{k,l}-\vec{w}\right\|}^{2}$

${\vec{u}}^{k+1}={\vec{u}}^{k,l}$

${\vec{w}}_{i}^{k+1}=\max\{(|D_{i}{\vec{u}}^{k+1}-\frac{{\vec{\lambda }}_{i}^{k}}{\beta }|-\frac{1}{\beta }),0\}\cdot \mathrm{sgn}(D_{i}{\vec{u}}^{k+1}-\frac{{\vec{\lambda }}_{i}^{k}}{\beta })$

$\begin{array}{l} {\vec{\lambda }}^{k+1}={\vec{\lambda }}^{k}+\beta (D{\vec{u}}^{k+1}-{\vec{w}}^{k+1})\\ {\vec{r}}^{k+1}={\vec{r}}^{k}-\beta \vec{g} \end{array}$

**end for**

A unique parameter setting was used for all one-dimensional denoising runs used for this manuscript. The parameter values for the above algorithm were as follows: $\mu =2^{-10},\beta =2^{-4},k=0,1,\ldots ,10,l=1,2,3,4,5,\sigma =0.$ For the algorithm as presented above, a convergence proof exists. We used *k = 10* as a termination criterion for the outer loop of the Algorithm, as it empirically provided sufficient precision for the fMRI BOLD time course denoising. We also observed that for our data, the precision of the algorithm was sufficient if a fixed number of steps, e.g., 5, were taken for the inner loop instead of the relative-error criterion; therefore, the inner iterations are forcibly stopped after five gradient steps.

#### 2.3.5 Echo restoration

The three temporal BOLD signal echoes were restored simultaneously for all voxels using the procedure described in Section 2.3.4 from echoes that were preprocessed only in the unwarp + realign step in SPM12, but—notably, for reasons explained in Section 2.2.2—not spatially smoothed.

### 2.4 T_2_^*^ estimation

From the restored (denoised) echoes, the dynamic T_2_^*^ time courses were estimated using the procedure described in Michálek et al. (2019), which is based on a weighted log-linear fit to a monoexponential model. The advantage of the said algorithm compared to usual monoexponential fitting procedures is a compensatory weighting of later echoes to account for noise boost caused by the logarithmic operation. Unlike in Michálek et al. (2019), for the dynamic T_2_^*^-TV estimation, we did not estimate the Rician noise floor since it would require more redundancy in the signal, e.g., a greater number of echoes. According to our experiences with only three noisy echoes, reliable estimation of three dynamically changing parameters (S_0_, T_2_^*^, and the noise floor) is not feasible. T_2_^*^ maps were calculated in individual subject spaces.

### 2.5 Methods of assessment of the BOLD signal quality obtained by TV-based denoising

We propose to quantitatively explore the efficiency of a denoising algorithm by comparing standard measures of fMRI signal quality between the outcome of the denoising and the signal before denoising, or between signals denoised using different methods.

We used two standard measures to quantify the BOLD signal quality:

- Temporal signal-to-noise ratio (tSNR) of the denoised *resting-state* BOLD signal (Friedman and Glover, 2006).
- Contrast-to-noise ratio (CNR) of the *task-induced* BOLD signal, as defined by Geissler et al. (2007).

The tSNR calculations were based on the nine resting-state datasets and the CNR calculations on the nine task datasets. Rather than comparing tSNR and CNR at individual voxels, we calculated their distributions over all nine study participants on subject-specific ROIs defined in Section 2.5.1. The efficiency of a denoising method was quantified by the value of the tSNR and CNR medians of the respective distributions. This method of denoising efficiency assessment is akin to that of Heunis et al. (2021). The difference is that we quantify the efficiency by calculating medians, which, to our experience, suppress outliers better than the means used by Heunis et al. (2021).

In addition, we defined, for task data, the following new measure of fMRI signal quality:

- Similarity between the particular type of task BOLD signal and the theoretical hemodynamic function (Section 2.5.4).

#### 2.5.1 Regions of interest

The temporal signal-to-noise ratio (tSNR) of the *resting-state* datasets was evaluated in *all* brain voxels indicated by a subject’s brain mask. The mask was generated subject-specifically using the histogram of the whole resting-state fMRI series. The histograms are bimodal, i.e., have two peaks. The background threshold was selected at the bin with the lowest histogram value between the two peaks. Voxels that violated the condition of a monotonic decrease of the multi-echo signal were excluded from the analysis.

To create well-defined ROIs for the nine *task* datasets, we selected participant-specific ROIs depending on the correlation between the second echo time courses and the theoretical hemodynamic response.

In fMRI analysis, multiple comparisons in different brain voxels are carried out simultaneously. If more than one *α*-level hypothesis test is performed, the risk of making *at least one* Type I (false positive) error will exceed α. This would result in an excessive number of false positives (Lindquist and Mejia, 2015). Therefore, some method of correcting for multiple comparisons is needed to identify areas of activity that reflect true effects and thus would be expected to be replicated in future studies (Han and Glenn, 2017).

The total number of simultaneous hypothesis tests (one for each voxel) for our nine participants ranged between 40,237 and 57,123. Unlike the frequently used Bonferroni correction or family-wise error rate (FWER) to guard against excessive false positives that arise from multiple comparisons, we used the Benjamini–Hochberg false discovery rate (FDR) correction procedure (Benjamini and Hochberg, 1995), which is more powerful than other methods that control false positive rates (Glickman et al., 2014). The prespecified FDR was *α* = 0.05, which means that, on average, 5% of the voxels found significant will be false positives. Participant-specific lists of voxels deemed active with *α* = 0.05 were generated using an FDR implementation available from https://www.mathworks.com/matlabcentral/fileexchange/71734-fdr-false-discovery-rate. For each study participant, their specific ROI was defined by their FDR-satisfying voxels.

To generate lists of voxels passing the FDR condition (i.e., the subject-specific ROIs), we worked out the following algorithm:

- For all voxels of the echo 2 of the task-induced subject data, calculate the normalized circular correlation ρ (cf. 2.5.4) between the fMRI time series and the normalized hemodynamic response of the task.
- For each voxel correlation, calculate the *t*-value as $t=\frac{\rho \sqrt{n-2}}{\sqrt{1-\rho }}$ (Cohen et al., 2013).
- For each voxel, calculate the Student’s *t* cumulative distribution function from the *t*-value using MATLAB’s function *tcdf*: P=tcdf(t,n−2) .
- From the voxel’s value of Student’s *t* cumulative distribution function, compute the p-value of the *t*-statistic as p=2⋅(1−P) .
- Using the FDR.m procedure, find indices of voxels that satisfy the condition FDR≤α with the significance level α = 0.05.

An example of one slice of the FDR-generated ROI is in Figure 3. The highest density of the ROI is seen in the visual cortex.

#### 2.5.2 tSNR calculation

The voxel-by-voxel tSNR values were calculated using the procedure proposed by Friedman and Glover (2006) and saved as one 3D array per dataset. The original MATLAB code for tSNR computation was kindly provided by Prof. Glover.

#### 2.5.3 CNR calculation

We adopted the CNR definition by Geissler et al. (2007):


$$\mathrm{CNR}=\frac{\mathrm{\Delta S}}{\sigma }$$

where ΔS is the average signal change (task-related variability, contrast), and σ is the non-task-related variability over time (time series noise), both calculated voxelwise. We calculated ΔS from the task data as the difference between the BOLD signal average across the task stimulation ON epoch and the average across the OFF epoch. First, we defined an *averaging* boxcar function synchronous with, but different from, the task *stimulation*. The averaging function had the value 1/(total number of ON points), where stimulation was ON, and [−1/(total number of OFF points)], where stimulation was OFF. The difference between the BOLD signal mean across the ON epoch and the mean across the OFF epoch was calculated as the maximum of the time lag-dependent cross-correlation function between the BOLD signal and the averaging boxcar function. Taking the maximum of the cross-correlation function eliminates the time lag between the stimulation and the hemodynamic response.

Since, for each participant, the *resting-state* runs were recorded using the same acquisition settings as the *task* runs, it is justified to assume that both had the same voxelwise standard deviation of the noise. Therefore, we used the voxelwise noise standard deviations σ that were obtained from the resting-state tSNR calculation to compute the voxelwise CNR.

#### 2.5.4 Similarity between the hemodynamic response function and the voxelwise BOLD signal time courses

We defined the similarity between the theoretical hemodynamic response and the BOLD signal time course as the maximum (with respect to the time shift) of the normalized circular cross-correlation function. The calculation involved the following steps:

- The *normalized* hemodynamic response (NHR, common for all voxels) was calculated by subtracting the mean from the *theoretical* hemodynamic response and rescaling the remaining zero-mean signal such that the sum of the squares of its time samples was 1.
- Analogously, the BOLD signals (either the T_2_^*-^TV-denoised time course, the *tedana.py*-denoised time course, the optimally weighted sum, or the T_2_^*^ raw time course) were normalized voxel by voxel.
- Calculation of the circular cross-correlation between the NHR and normalized BOLD functions, which amounts to repeated calculation of the scalar product of unity vectors. The magnitude of the normalized circular cross-correlation thus lies between 0 and 1. The value of 1 indicates that the NHR and the normalized BOLD signal are identical.

Detecting the maximum of the circular correlation function has an advantage over a simple correlation coefficient in that it eliminates the time lag between the stimulation and the BOLD response if there is such.

#### 2.5.5 Statistical evidence for observations made on the output data

fMRI is a dynamic process that yields individual time series data for tens of thousands of brain voxels with tens of thousands of different tSNR, CNR, or correlation values. The efficiency of two fMRI methods can be compared by computing some parameters of the tSNR, CNR, or correlation, like the median or the mean value. To decide if some method (e.g., TV-based denoising) behaves differently from another method (e.g., NORDIC denoising), we can calculate the probability that a difference in some parameter values, e.g., means, is not random.

It turned out that the probability distribution of the tSNR, CNR, or correlation values is not normal (Gaussian). Therefore, we are using for hypothesis testing the non-parametric Wilcoxon rank sum test implemented in MATLAB.

## 3 Results

### 3.1 Example: TV-based 1D restoration of multi-echo resting-state BOLD signal in one voxel

In Figure 4, the original (red) noisy 3-echo time courses from 312 resting-state fMRI frames are compared with their TV-restored (green) counterparts for the same green-marked voxel as in Figure 1. As required for smooth T_2_^*^ estimation, the random noise in the green signals has been, to a large extent, removed by minimization of the TV. In contrast to Figure 2, where the means of the echo time courses were pushed up or down, the local signal mean values of all three echoes in Figure 4 have been preserved thanks to the data fidelity term of TV-based restoration.

### 3.2 Example: resulting T_2_^*^ maps from TV-restored echoes

The TV-based restoration of temporal fMRI time courses was applied to the multi-echo task-related fMRI datasets described in Section 2.1. From the TV-restored echo time courses, dynamic T_2_^*^ maps were estimated as described in Section 2.4. Figure 5 shows examples of signal restoration and dynamic T_2_^*^ mapping in three different voxels.

#### 3.2.1 Sample voxels

In Figure 5, examples of raw and restored BOLD echo time courses from three brain voxels are compared, and the estimated dynamical T_2_^*^ waveforms are shown.

The three sample voxels were selected based on their response to a visual task execution. For each voxel of the brain with a valid dynamic T_2_^*^ time series, we evaluated its normalized cross-correlation, that is, its similarity, with the theoretical hemodynamic response. The theoretical hemodynamic response was calculated by convolving the task “on”/“off” boxcar function with the canonical hemodynamic response function (HRF) and is plotted in arbitrary units. We used the canonical HRF calculated as the difference of Gamma functions and implemented as a MATLAB function named spm_hrf.m in the SPM12 package (available from https://www.fil.ion.ucl.ac.uk/spm/software/download/). The two time courses were normalized such that, if identical, the normalized cross-correlation was 1. We sorted the brain voxels in descending order of the cross-correlation. For illustration in Figure 5, we selected two voxels with high correlation, (29,7,25) and (42,28,39), as well as one voxel showing no obvious similarity with the hemodynamic response (24,38,24). Voxel (29,7,25) was in the visual cortex, and voxel (42,28,39) was in the motor cortex.

The restored echoes (in green) together with the raw echoes (in red) in panels (B,E,H) confirm that the procedure detailed in Section 2.3.4 yields restored echo signals that satisfy the property of the BOLD signal discussed in Section 2.3.1: in accordance with invasive BOLD signal measurements in animals, the restored echoes change smoothly. Furthermore, the restored echoes satisfy the requirement essential for accurate T_2_^*^ estimation: they preserve the local mean (mean value in a small time neighborhood) of the raw echoes, as required in Section 2.2.2.

The dynamic T_2_^*^ time courses in the three sample voxels are plotted in green in Figures 5C,F,I. For visual comparison, the theoretical response is plotted in blue below them. Similar to the restored echoes, the estimated T_2_^*^ waveforms are smooth. The echoes, the T_2_^*^, and the hemodynamic response waveform had the same sampling time, i.e., TR = 1,800 ms.

We dismissed the highest T_2_^*^ contrast that was found in the voxel (31,7,24). The peak-to-peak amplitude measured in the MATLAB figure was approximately 21 ms, but such a high value suggested that it was located in a large blood vessel rather than in the visual cortex.

Synchronicity between the stimulation, the measured BOLD signal, and the associated T_2_^*^ variations was also detected in motor cortex voxels such as (42,28,39) in panels (D,E,F).

Some brain regions are not activated by visual stimulation. For example, the voxel (24,38,24) depicted in panels (G,H,I) lies on the transition between the gray and white matter and should not be affected by the visual task that induced blood oxygenation in the visual cortex. This is confirmed both by the time courses of the three echoes at that voxel (H) and by the resulting time course of the T_2_^*^ estimate (I, green) that does not respond to stimulation.

#### 3.2.2 tSNR (temporal signal-to-noise ratio) of resting-state BOLD echoes evaluated over nine volunteers

To quantify differences between the TV-based denoising and known state-of-the-art denoising methods, we calculated the distributions of tSNR values of the unprocessed echo 2 in the resting state and several denoised variants of the BOLD signal processed by the denoising code made publicly available by its authors. For each of the nine *resting-state* datasets reported in 2.1, we compared the distributions of voxelwise tSNR values of the following eight BOLD signal variants (Figure 6):

A) TV-denoised echo 2 using the novel TV-based time series denoising algorithm presented in this article (*TV-l2*).
B) Echo 2 denoised using the NIFTI_NORDIC code by Vizioli et al. (2021) (available at https://github.com/SteenMoeller/NORDIC_Raw). We obtained the best results with default parameter settings.
C) Echo 2 despiked using the *3dDespike* function of the Analysis of Functional NeuroImages (AFNI), a software suite for the analysis and display of multiple MRI modalities, code available at: https://afni.nimh.nih.gov/, with the best parameter settings corder = 25, cut = (1.2,1.7).
D) Unprocessed echo 2 of the respective subject’s resting-state dataset.
E) T_2_^*^ mapping from three echoes using the TV-based workflow described in this study.
F) *tedana*-three-echo-denoised BOLD signal (Kundu et al., 2012), code available at: https://github.com/ME-ICA/tedana, script *tedana.py*. We obtained the best results with the static T_2_^*^ log-linear fit and the PCA method “kundu-stabilize.”
G) “optimal combination” of three echoes as described by Posse et al. (1999), code implemented in the signal preprocessing part of the *tedana* package, at: https://github.com/ME-ICA/tedana, script *t2smap.py*.
H) T_2_^*^ mapping from three raw echoes without any denoising, also computed by *tedana*, script *t2smap.py*.

As mentioned in Section 2.1, for each of the nine participants, one task and one resting-state run were measured with identical acquisition settings. This allowed us, per each resting-state run, to calculate and save a 3D array of the voxel-by-voxel standard deviations σ of the BOLD signal residuals after second-order polynomial detrending and use it as a noise estimate for the subsequent calculation of the temporal CNR in Section 2.5.3.

The resulting tSNR histograms shown in Figure 6 were calculated across all brain voxels of all participants. Figure 6 compares the tSNR distributions of the raw echo 2 and seven different preprocessing methods applied to the resting-state BOLD signal time courses. Depending on whether researchers consider single-echo or multi-echo fMRI data, the left or the right column of Figure 6 may be more relevant. tSNR medians and their respective confidence intervals are included with each plot. The *TV-l2* denoising of echo 2 (A) produces the highest tSNR of the four single-echo BOLD signal processing results, both in the median and in the upper tail of the distribution. The single-echo NORDIC-denoised time course features better tSNR distribution (B) than the 3D-despiked echo (C). The 3D-despiked echo 2 using AFNI’s *3dDespike* function has, in turn, a somewhat better signal-to-noise ratio than the raw echo 2 (D), which is often used for fMRI data analysis and can be regarded as a reference BOLD signal.

The right column shows that multi-echo preprocessing of fMRI data does not necessarily yield higher tSNR values. The *tedana* package (F) has the highest tSNR of the multi-echo methods, yet it is outperformed by the single-echo methods (A) and (B). tSNR of the T_2_^*^ mapping from TV-denoised three echoes (E) is for resting-state data, about on a par with the weighted sum of three echoes, referred to as “optimal combination” (G). An important difference, however, is that T_2_^*^ mapping delivers quantitative output measured in time units (seconds), while the output of *tedana* or optimal combination is in arbitrary units. The “optimal combination,” essentially a weighted sum of three echoes, acts as a low-pass filter, and its tSNR is better than that of the plain unprocessed echo 2 (H). It may still be attractive since it is computationally less demanding than T_2_^*^ mapping or *tedana*, but it is also less efficient.

It should be pointed out that, contrary to single-echo data that are always in arbitrary units, the two multi-echo methods of T_2_^*^ mapping, either from raw echoes or from TV-denoised echoes, are quantitative in nature since they provide results in the physical units of time. For some applications, like T_2_^*^ weighting of fMRI signals, this is a requirement.

We tested the statistical significance of the differences in tSNR distributions of preprocessed fMRI signals using the Wilcoxon rank sum test. The results are summarized in Section 3.2.7.

#### 3.2.3 Comparison of echoes processed by different denoising algorithms at a particular voxel

To convey a visual impression of how denoising modifies the echo time series, in Figure 7, we plotted the results of different denoising approaches at the voxel (32,6,23) of the resting-state run with our subject sub-01. Voxel (32,6,23) was selected because it had the highest correlation between the task-induced T_2_^*^ time series and the hemodynamic response function. Three denoised versions of echo 2 are compared with the measured, unprocessed echo 2.

A) TV-denoised echo 2 using the TV-based time series denoising algorithm.
B) Echo 2 denoised using the *NIFTI_NORDIC* code by Vizioli et al. (2021).
C) Echo 2 despiked using the *3dDespike* function of the AFNI software suite.
D) Unprocessed echo 2 at (32,6,23) of sub-01.

tSNR values of the echo 2 variants are printed in the lower right corner of the plots.

For participant sub-01 at voxel (32,6,23), the TV-denoised resting-state echo 2 yields the highest tSNR and is—contrary to the other time series—smooth. The second-best tSNR was generated by AFNI’s *3dDespike* function. tSNRs of (B), (C), and (D) were not much different.

#### 3.2.4 CNR of four single-echo and four multi-echo preprocessing methods of task BOLD data

Posse et al. (1999) presented a theory concerning multi-echo signal processing strategies aimed at enhancing the BOLD contrast sensitivity. Posse’s theoretical analysis showed that weighted summation of multiple echoes (later referred to as “optimal combination”) could provide a 2.35-fold gain in CNR compared with the corresponding single-echo measurements. Another multi-echo processing approach, the T_2_^*^ fitting of the multi-echo data, was predicted by Posse et al. (1999) to yield a gain in sensitivity, although approximately 10% less than the weighted echo combination.

We first calculated CNR distributions for the single-echo fMRI *task data* by applying methods that we previously used for the tSNR analysis on *resting-state data* (Section 3.2.2):

A) TV-denoised echo 2 using the novel TV-based time series denoising algorithm presented in this article (*TV-l2*).
B) Echo 2 denoised using the NIFTI_NORDIC code by Vizioli et al. (2021).
C) Echo 2 despiked using the *3dDespike* function of the AFNI software.
D) Unprocessed echo 2 from the resting-state dataset of the respective subject.

Then, we compared the CNR achieved by the new TV-denoised multi-echo T_2_^*^ mapping with that of the two multi-echo BOLD data-processing methods analyzed by Posse et al. (1999) and the *tedana.py*-implemented multi-echo denoising by Kundu et al. (2012):

E) T_2_^*^ mapping from three echoes using the TV-based workflow described in this study.
F) *tedana* multi-echo denoising, script *tedana.py*.
G) Weighted multi-echo summation. To calculate the weighted echo summation (Posse et al., 1999) for all voxels, we used the script *t2smap.py* of the freely available software *tedana* (DuPre et al., 2021).
H) T_2_^*^ mapping from three raw echoes without any denoising, also computed by *tedana*, script *t2smap.py*.

CNR was compared for the total of nine participants’ task data on subject-specific, FDR-generated ROIs (see Section 2.5.1).

The left column of Figure 8 compares CNR distributions over all *task* runs of nine study participants for four types of *single-echo* task-based BOLD signal processing: (A) The *TV-l2* denoising of echo 2 produces the highest CNR of the four single-echo BOLD signal processing methods, (B) the CNR distribution achieved by single-echo NORDIC denoising is higher than that in (C), where the CNR histogram for the 3D-despiked echo is shown. The 3D-despiked echo 2 has a better CNR than the raw echo 2 (D), which is frequently used for fMRI data analysis.

The right column of Figure 8 shows that the T_2_^*^ estimate from three *TV-l_2_* denoised echoes (E) has the highest CNR median of the four methods, with many non-zero CNR counts in the upper tail of the histogram. The *tedana.py* denoised BOLD signal from three task echoes (F) is the second best in multi-echo methods. In (G), the CNR of the “optimal combination” is slightly lower. CNR of the T_2_^*^-raw estimate from three *raw* echoes (H), although computationally more expensive, is even lower than that of the optimal combination because—due to lack of denoising—it amplifies noise. The low CNR values in Figure 8D could also be expected from the low similarity of raw T_2_^*^ estimates with the theoretical hemodynamic response in Figure 9E.

The results of a statistical test of significance for the CNR medians are presented in Section 3.2.7.

#### 3.2.5 Similarity between the theoretical HRF and the voxelwise multi-echo-based BOLD signal time courses

Figure 9 shows, for each of the four multi-echo-based BOLD signal variants, the voxel time course that best matched the shape of the NHR. The noise superimposed on the waveforms (C) through (E) violates the assumption of smooth blood oxygenation changes. The T_2_^*^-*TV* time course is smooth as required. The smoothness of the T_2_^*^-*TV* signal is also reflected in the best values of the NHR-BOLD signal correlation.

Figure 9 depicts only the results from the first volunteer. To verify that the T_2_^*^-TV method brings about statistically significant improvements, we further performed statistical tests on data from all study participants. As the distribution of correlation values is bounded to [−1,1], it was not possible to check for differences in the correlation averages using the t-tests or ANOVAs, because they assume the data to be normally distributed. We instead tested the hypothesis that the best correlation values are samples from continuous distributions with equal *medians*, against the alternative that they are not. This was performed using the non-parametric Wilcoxon (or *ranksum*) test. In three pairwise Wilcoxon tests, we compared the median of the best correlation values across all study participants between the new T_2_^*^-TV method and another well-established method of multi-echo BOLD signal preprocessing. Significance level of the tests was *α* = 0.05. We calculated the Wilcoxon tests using the MATLAB *ranksum* function. Table 1 summarizes the results.

**Table 1:** Results of the Wilcoxon right-tailed rank sum test with a 5% significance level.

Comparison between	Median and interquartile range (IQR) of the best correlation of T_2_^*^-TV over nine subjects	Median and interquartile range of the best correlation of the compared method over nine subjects	*p*-value	Is the median of the best T_2_^*^-TV correlations of nine subjects significantly higher?
T_2_^*^-TV vs. tedana	Median = 0.966 IQR = 0.0062	tedana: median = 0.925 IQR = 0.0357	0.000144	Yes
T_2_^*^-TV vs. optimal combination		Optimal combination: median = 0.941 IQR = 0.0275	0.000617	Yes
T_2_^*^-TV vs. T_2_^*^-raw		T_2_^*^ raw: median = 0.921 IQR = 0.0364	0.000082	Yes

All *p*-values are less than 0.05, which allows us to accept the alternative hypothesis that, for the nine test subjects, the median of the best correlation values between the T_2_^*^-TV fMRI signal and the normalized hemodynamic response is significantly higher than the correlation medians obtained for other multi-echo BOLD signal processing methods.

The low *p*-values indicate that, for the T_2_^*^-TV method, the highest median of the best correlation values over all subjects is statistically significant.

The results of Table 1 regarding medians and interquartile ranges are presented visually in Figure 10.

#### 3.2.6 The effect sizes

To quantify the relationship between the visual stimulation and the BOLD signal response at a voxel, we chose the normalized circular cross-correlation function defined in Section 2.5.4. It has the advantage that the magnitude of its values lies between [0,1], so there is no need for an additional normalization or for a special treatment of outliers. Hence, distribution means can be calculated instead of medians, which sometimes leads to shorter computation times. For a perfect match between the normalized theoretical stimulation and the normalized BOLD response, i.e., the maximum effect size, the value of normalized cross-correlation is 1. It is also easily computed by means of standard numerical program packages without the need to delve into more specialized software. We evaluated the correlation over all brain voxels identified by subject-specific brain masks.

Illustrations for the same four single-echo and four multi-echo preprocessing methods that we applied to the preprocessing of fMRI task data are summarized in Figure 11, which displays histograms of the mean normalized correlation between the normalized theoretical hemodynamic response to stimulation and the normalized outcome of four single-echo and four multi-echo fMRI preprocessing methods. The NORDIC denoising in panel (A) yields the highest stimulus–response correlation, with the distribution for TV-based echo denoising (B) visually almost indistinguishable. All three single-echo denoising methods, including 3dDespike (C), improve correlation substantially compared to (D), the unprocessed echo 2.

TV-based T_2_^*^ mapping (E) yields the best correlation among the multi-echo methods and provides quantitative results, contrary to other methods that yield outputs in arbitrary units. *tedana* (F) follows closely. For the “optimal combination” (G), the effect size is surprisingly low—the mean value is slightly higher than that of the unprocessed single-echo signal (D). T_2_^*^ mapping from raw echoes (H) deteriorates the correlation, probably due to noise amplification through the log-linear fitting.

Figure 12 offers another insight into the effect sizes. It displays, for all four single-echo and all four multi-echo methods, voxels whose correlation with the theoretical response to stimulation is greater than 0.5, sorted in descending order. The intersection of each curve with the horizontal axis shows how many such voxels were produced by the respective method. Obviously, single-echo NORDIC denoising and the novel TV-based echo denoising described here produced the maximum of high-correlation voxels. This coincides with the good agreement between the histograms in Figures 11A,B.

#### 3.2.7 Wilcoxon test for comparing the medians of the tSNR, CNR, and correlation distributions

tSNR values for preprocessed fMRI *resting-state* data (Figure 6) and CNR and correlation values for preprocessed *task data* (Figure 8 and 11, respectively) are always ≥0 . Thus, their distributions are not normal and do not satisfy the assumptions for the t-test. For this reason, we used the Wilcoxon rank sum test implemented in MATLAB. The results are summarized in Table 2.

**Table 2:** Results of the Wilcoxon right-tailed rank sum test for comparing the tSNR, CNR, and correlation medians of four *single-echo* and four *multi-echo* fMRI preprocessing methods with a 5% significance level for the hypothesis test that median 1 is significantly higher than median 2.

Method 1	Median 1	Interquartile range 1	Method 2	Median 2	Interquartile range 2	*p*-value	Significance h: median 1 is significantly higher than median 2
tSNR of single-echo methods for resting-state data							
TV_Echo2	154.5	119.25	NORDIC_Echo2	95.85	55.408	0	1
			3dDespike_Echo2	58.19	29.918	0	1
			Echo2	46.51	23.896	0	1
tSNR of multi-echo methods for resting-state data							
tedana	76.23	36.934	T2star-TV	68.37	52.733	0	1
			OptCom	69.99	33.686	0	1
			T2star-loglin	19.25	10.147	0	1
CNR of single-echo methods for task data							
TV_Echo2	1.1463	0.8861	NORDIC_Echo2	1.115	0.72726	0.39251	1
			3dDespike_Echo2	0.839	0.48016	0	1
			Echo2	0.679	0.39251	0	1
CNR of multi-echo methods for task data							
T2star-TV	0.85982	0.9289	tedana	0.799	0.61959	0	1
			OptCom	0.759	0.54445	0	1
			T2star-loglin	0.486	0.43889	0	1
Normalized correlation of single-echo methods for task data							
NORDIC_Echo2	0.19445	0.15674	TV_Echo2	0.182	0.16837	0	1
			3dDespike_Echo2	0.18582	0.13239	0	1
			Echo2	0.12396	0.085902	0	1
Normalized correlation of multi-echo methods for task data							
T2star-TV	0.19559	0.17262	tedana	0.17425	0.15328	0	1
			OptCom	0.12996	0.095696	0	1
			T2star-loglin	0.11921	0.072266	0	1

The method with the highest median was always selected as method 1. All *p*-values are less than 0.05, which allows us to accept the alternative hypothesis that, over all nine test subjects, the median of method 1 is significantly higher than that of method 2.

## 4 Discussion

The multi-echo data acquisition protocol is different from standard relaxometric protocols, and multi-echo fMRI data are noisy. If T_2_^*^ maps are estimated from a low number of echoes, the fitting algorithm may even amplify the noise, as can be verified both by a simple analysis [Michálek et al., 2019, Equations (12–14)] and empirically. Therefore, efficient denoising is vital for low-noise T_2_^*^ mapping.

It is known that BOLD signal time courses measured *in vivo* are smooth. Based on this physiological BOLD signal property, we proposed a novel *denoising* algorithm to render the denoised signals *smooth* while keeping them as close as possible to the raw measured signal. These two requirements can be cast into a mathematical problem to be solved:

For each raw measured voxel BOLD time series, calculate a reconstructed time series such that the following two requirements are kept in balance:

- Minimize the TV of the oscillations in the time series.
- Make the *l_2_* norm of the difference between the measured time series and the reconstructed time series as small as possible.

For this particular formulation of the problem of denoising, we proposed a novel TV-based *TV-l_2_ minimization algorithm* for time series denoising. The algorithm removes, from a measured time series, disturbances up to a certain duration and magnitude (Chambolle and Pock, 2016). Such disturbance removal is not linked to any particular frequency band.

We compared our TV-denoised time series with the results of denoising obtained by other state-of-the-art fMRI denoising methods listed below. We used publicly available code implemented by the methods’ authors. Each of the denoising methods solved another problem formulation; therefore, the results of denoising were also different.

- *3dDespike* of the AFNI software suite for the analysis of multiple MRI modalities^1^ matches a fixed-order Fourier series to the voxel time series and then nonlinearly (using the tanh function) suppresses the difference between the curve and the data time series (the residuals) to minimize the median absolute difference (MAD).
- *NORDIC* (Vizioli et al., 2021) removes from the time-signal principal components (PCs) that cannot be distinguished from zero-mean Gaussian distributed noise.
- *tedana.py* (Kundu et al., 2012; Kundu et al., 2017) first removes some PCA components from the time series and then carries out ICA, in which independent components (ICs) are labeled as “BOLD” or “non-BOLD” with “non-BOLD” regarded as noise and eventually removed from the cleaned signal.

The raw BOLD signal contains many isolated spikes that disappear after a few sampling periods. Isolated pulses in the time domain contain all frequencies because the Fourier transform of a single spike contains all frequencies with the same magnitude. Therefore, random signal spikes cannot be removed using frequency filters like Gaussian smoothing, moving average, low-pass, band-pass, high-pass, etc., that suppress only a subset of frequencies in the *frequency domain*. An attempt to remove isolated peaks present in multi-echo fMRI measurements using, e.g., low-pass filters *will* result only in making the peak wider and lower. Similarly, removing whole time series (“component”), as done by Fourier, PCA, or ICA denoising methods, generally does not remove isolated spikes.

The superior tSNR and CNR values of the TV-based denoising demonstrated in Section 3.2 are due to the fact that the TV-based minimization algorithm is capable of removing disturbances that are isolated in *time* (Chambolle and Pock, 2016), whereas denoising approaches based on principal component removal (*NORDIC* or *tedana.py*) or independent component removal (*tedana.py*) remove whole time series. PC- and IC-based denoising approaches lack the ability to remove isolated *time events*.

Of the three available denoising code implementations that we tested, only *3dDespike* possesses the ability to suppress isolated signal pulses. Unfortunately, the available code has built-in constraints, which we were not able to override when optimizing the algorithm’s parameters.

We also tried to use a 1D wavelet-based denoising code for resting-state fMRI time series (Patel et al., 2014) for our block-design task data. Unfortunately, it did not work since, in activated voxels, the algorithm removed the whole width of stimulation-synchronous pulses from the BOLD signal.

One pitfall to be avoided in T_2_^*^ mapping is the fact that Gaussian spatial smoothing, a standard step in fMRI signal processing, irreversibly destroys the mean values of the echo time courses due to blending neighboring voxel values: e.g., echo 1 time series can be shifted down while echo 2 and echo 3 stay at their means. The shift of any of the echoes irreparably distorts the T_2_^*^ estimates; hence, T_2_^*^ mapping *after* spatial smoothing is unreliable. This was the reason why we created the T_2_^*^ maps based on data that were only unwarped and realigned before TV-based denoising.

T_2_^*^ time courses estimated from TV-denoised echoes yielded a quantitative BOLD signal (i.e., measurable in time units) that was smooth, as implied by invasive BOLD signal measurements in animals, and matched the theoretical hemodynamic response better than other BOLD signal variants commonly used in fMRI analysis (echo 2, *3dDespike*-d echo 2, optimal combination or T_2_^*^ fit from the raw echoes, ME-ICA denoised multiple echo signals, or *NORDIC*-denoised time courses). In terms of BOLD signal quality metrics, T_2_^*^ time courses calculated from *TV-*denoised echoes showed, in the median, higher tSNR (except tedana) and higher CNR than the other BOLD signal forms.

Another observation we made was that the detected T_2_^*^ amplitude (the contrast ΔS ) is diminished through some other preprocessing stages, e.g., spatial normalization to the MNI space. This is not surprising since spatial normalization (i.e., image registration) is necessarily accompanied by voxel interpolation, averaging, or deletion. Therefore, we recommend performing T_2_^*^ mapping immediately after unwarping/realigning the data and before normalization to MNI space. However, even then, it may be necessary to use a dedicated registration algorithm that will guarantee not to destroy the dynamic T_2_^*^ maps through spatial normalization to MNI.

## 5 Conclusion

The aim of this study was to develop a fast and robust algorithm for voxelwise time-dependent T_2_^*^ mapping based on multi-echo (three-echo) fMRI data. The T_2_^*^ maps could be used, e.g., for derivation, from the whole time series, of static quantitative parameters like mean T_2_^*^ or delta T_2_^*^ (corresponding to BOLD percentage signal change) to enable robust estimation of tissue properties. Such characteristics can be subsequently verified, e.g., on an epileptic dataset from the perspective of the possibility to detect epileptic lesions.

We addressed the problem of noise amplification in T_2_^*^ estimates from noisy echoes by deriving a time series reconstruction algorithm based on the revolutionary image-denoising approach by Rudin et al. (1992). This enabled us to obtain voxelwise smooth quantitative T_2_^*^ maps of the whole brain featuring higher BOLD signal quality than the “optimal combination” widely used in the fMRI analysis or than other advanced BOLD data-processing methods. The minimalistic number of a mere three echoes fosters rapid data acquisition and extends the range of applications where knowledge of dynamic T_2_^*^ maps may help research or therapy.

## Data Availability

The raw data supporting the conclusions of this article will be made available by the authors, without undue reservation.

## Footnotes

^1^ https://afni.nimh.nih.gov/pub/dist/doc/htmldoc/programs/alpha/3dDespike_sphx.html
